# Asparaginase enhances CAR-T cell antitumor immunity by asparagine metabolic reprogramming and central memory induction in ALL

**DOI:** 10.1016/j.ymthe.2025.08.019

**Published:** 2025-08-12

**Authors:** Xinting Zhu, Leng Han, Dingyuan Bai, Lei Yi, Yonghong Zhao, Shuaibing Liu, Run Gan, Bo Xin, Yixing Tu, Jianping Zhang, Yonglong Han, Juan Hao, Zixue Xuan, Cheng Guo, Quanjun Yang

**Affiliations:** 1 Department of Pharmacy, Shanghai Sixth People’s Hospital Affiliated Shanghai Jiao Tong University School of Medicine, Shanghai 200233, China; 2 Department of Burn, Ruijin Hospital, Shanghai Jiao Tong University School of Medicine, Shanghai 200025, China; 3 Department of Surgery, Shanghai Sixth People’s Hospital Affiliated Shanghai Jiao Tong University School of Medicine, Shanghai 200233, China; 4 Department of Pharmacy, the First Affiliated Hospital of Zhengzhou University, Zhengzhou 450052, China; 5 Department of Endocrinology, Shanghai TCM-Integrated Hospital Affiliated to Shanghai University of Traditional Chinese Medicine, Baoding 230, Hongkou 200086, Shanghai, China; 6 Center for Clinical Pharmacy, Cancer Center, Department of Pharmacy, Zhejiang Provincial People’s Hospital (Affiliated People’s Hospital), Hangzhou Medical College, Hangzhou 310014, Zhejiang, China

**Keywords:** asparagine synthetase, asparaginase, asparagine, chimeric antigen receptor, immunotherapy, leukemia, acute lymphoblastic leukemia, cancer metabolism, metabolic reprogramming, antitumor immunity

## Abstract

High levels of asparagine synthetase (ASNS) in acute lymphoblastic leukemia (ALL) lead to immunotherapy resistance. Our study showed that ASNS overexpression (OE) in NALM6-GL cancer cells attenuated chimeric antigen receptor (CAR)-T cell-mediated cancer cell lysis. Asparaginase (ASPG) is an approved drug that breaks down circulating asparagine in leukemia cells, thereby depriving cancer cells of asparagine and inhibiting cancer growth. We proposed a hypothesis that ASPG-engineered CAR-T cells undergo phenotype switching to overcome immunotherapy resistance in ALL. Coculture killing assay showed ASPG-OE CAR-T cells exhibited increased killing efficacy against ASNS-OE cancer cells by enhancing the expression of granzyme B, interferon gamma, and tumor necrosis factor alpha, whereas ASPG-knockout (KO) CAR-T cells showed decreased cancer cell lysis efficiency. Phenotypic analysis revealed that ASPG-OE CAR-T cells exhibited distinct phenotypes, including increasing central memory T cells percentage, while decreasing effector memory T cells and effector memory cells that re-expressed CD45RA cells proportions. This distinct phenotype switch of ASPG-OE CAR-T cells toward central memory T cells exerted the increased killing efficacy against NALM6-GL cells even without ASNS-OE. The *in vivo* xenograft mouse model confirmed that ASPG-OE CAR-T cells exhibited superior anticancer activity against NALM6-GL cancer cells, while ASPG-KO CAR-T cells exhibited inferior anticancer activity. Taken together, ASPG orchestrates CAR-T cell distinct phenotype toward central memory T cells and reprogramming of asparagine metabolism for enhancing antitumor immunity.

## Graphical abstract

**Figure:**
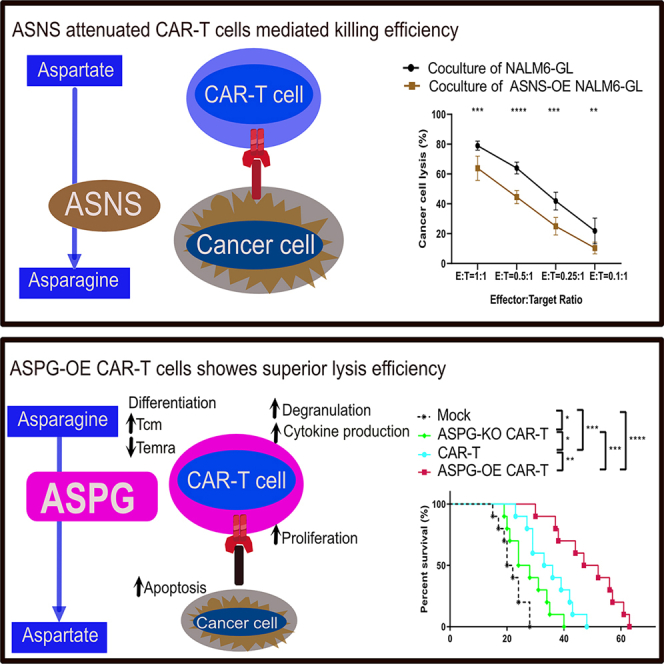

## Teaser

Deprivation of asparagine in cancer cells inhibits cancer growth, while asparagine synthesis in acute lymphoblastic leukemia leads to immunotherapy resistance. Asparaginase overexpression in CAR-T breaks down circulating asparagine and enhances antitumor immunity. ASPG orchestrates CAR-T cell distinct phenotype toward central memory T cells for long-term protection against cancer cells.

## Introduction

The prognosis and treatment of acute lymphoblastic leukemia (ALL) have significantly improved in recent decades owing to the development of effective intensified chemotherapy protocols for children and young adults.^1^^,^^2^ However, the outcomes in older adults and patients with relapsed or refractory ALL remain poor.^3^ Asparaginase (gene: *ASPG*) has been clinically used as a crucial and essential component of chemotherapy for ALL. Treatment with ASPG depletes the circulating nonessential amino acid asparagine in leukemia cells, thereby inhibiting leukemia cell growth and inducing cell death.^4^ Ongoing research continues to find ways to maximize the benefits of ASPG-based treatment while minimizing potential adverse effects.^5^ Clinically, hypersensitivity, silent inactivation, and relapse risk often limit the long-term usage of ASPG products.^6^ Recent advancements in adoptive immunotherapy with chimeric antigen receptor (CAR)-modified T cells have shown promise in treating pediatric and adult patients with relapsed/refractory ALL.^7^ However, high levels of asparagine synthetase (ASNS) in hematologic malignancies lead to resistance to immunotherapy, including CAR-T cell therapy.^8^^,^^9^

CAR-T cells are created by isolating T cells and modifying them with lentiviral vectors for the integration of CARs into the host genome with distinct phenotypes and functional features.^10^ CARs are constructed by combining functional sequence domains from different subunits of immunologically active proteins, including a single-chain variable fragment (scFv) domain generated from the variable heavy and variable light domains of an antigen (such as CD19), a hinge region, a transmembrane domain, an intracellular signaling domain, a costimulatory domain (CD28 or CD137), and a CD3-zeta activation domain. These CAR-T cells are capable of targeting cancer cells bearing a specific antigen for destruction through active cytolysis and indirect immune effector mechanisms triggered by the production of cytokines such as interferon gamma (IFN-γ) and tumor necrosis factor alpha (TNF-α).^11^ However, the efficacy of CAR-modified T cells in treating relapsed/refractory ALL has been limited because of the complex heterogeneity of cancer and the functional phenotype of CAR-T cells.^12^ The functional domains of different immunologically active proteins regulate the CAR-T-cell phenotype and function.^13^ Previous studies have explored synthetic biology methods, such as altering costimulation,^14^ coupling immunologically active proteins,^15^ and reprogramming CAR-T-cell metabolism,^16^^,^^17^^,^^18^ to increase CAR-T-cell activity.

In this study, we propose the usage of synthetic biology technology to couple the asparaginase gene *ASPG* with CAR-T cells for the treatment of ALL through phenotypic and functional reprogramming. Along with transcription and translation of chimeric antigen receptors, the *ASPG* gene functions as the enzyme ASPG by breaking asparagine into aspartate, depleting serum asparagine, and effectively starving leukemia cells.^19^ Asparagine is a nonessential amino acid that leukemia cells are unable to produce and therefore rely on external sources.^20^ The depletion of asparagine leads to reduced DNA, RNA, and protein synthesis, ultimately resulting in the death of leukemia cells. Interestingly, CD8^+^ T cells exhibit a biphasic response to asparagine restriction, maximizing their metabolic fitness and antitumor functionality.^21^ Asparagine is produced intracellularly by ASNS.^22^ When naive CD8^+^ T cells were stimulated by T cell receptor (TCR) and CD28 in asparagine-depleted medium *in vitro*, they showed reduced cell proliferation and cytokine production due to low or absent ASNS expression.^23^ However, after 24 h of activation, TCR induced mechanistic target of rapamycin kinase (mTOR)-dependent signaling and led to the upregulation of ASNS, enabling CD8^+^ T cells to function independently of extracellular asparagine.^24^ This finding indicates the importance of asparagine uptake and ASNS expression for the activation and functional features of CD8^+^ T cells. Our study aimed to explore the phenotypic and functional characteristics of ASPG-coupled CAR-T cells for the treatment of ALL.

## Results

### ASNS overexpression attenuated CAR-T-cell-mediated cancer cell lysis

**Figure 1:**
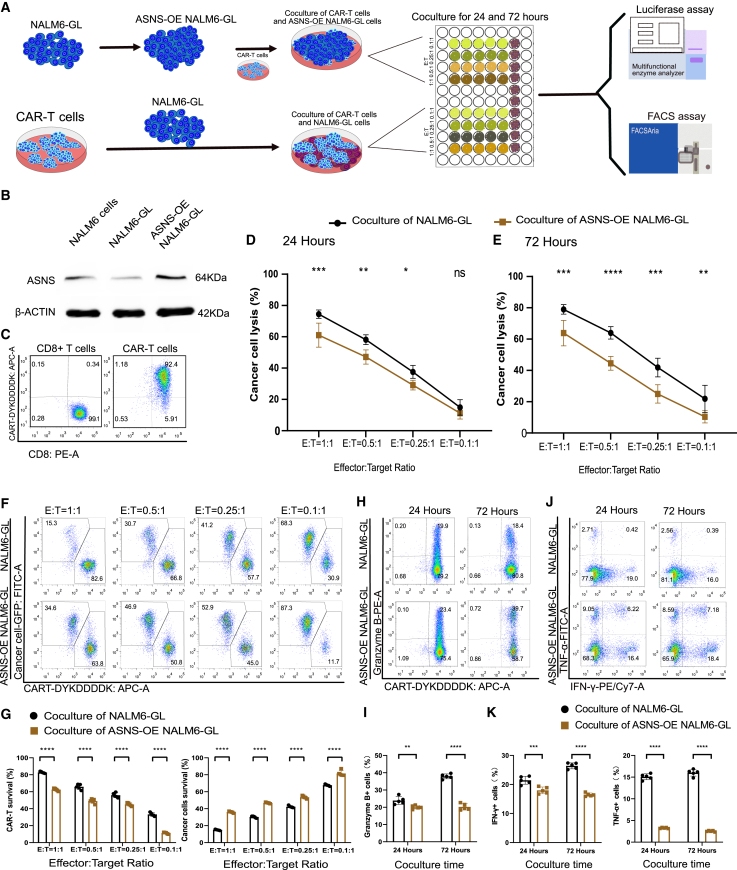
ASNS overexpression attenuated CAR-T cell-mediated cancer cell lysis efficiency (A) Flowchart for the coculture of CAR-T cells with ASNS-OE NALM6-GL cancer cells and functional assay of killing potential. (B) Immunoblotting results showing ASNS protein expression in ASNS-OE NALM6-GL cancer cells and control cells. (C) CAR expression in anti-CD19 CAR T cells was produced through lentivirus spin infection and flow sorting enrichment. (D and E) Cancer cell lysis assay of effector CAR-T cells to target cancer cells (E:T) at ratios of 1:1, 0.5:1, 0.25:1, and 0.1:1 after coculture for 24 h (D) and 72 h (E). Two-way ANOVA was used for statistical analysis, and Sidak’s multiple comparisons test was used for comparisons between the two groups. *p* values are denoted with asterisks as follows: not significant (ns); ∗*p* value < 0.05; ∗∗*p* value < 0.01; ∗∗∗*p* value < 0.001; and ∗∗∗∗*p* value < 0.0001. (F and G) Representative flow cytometry pseudocolor map (F) and grouped histogram (G) showing the resident CAR-T cells and cancer cells at an E:T ratio of 1:1 after coculture for 72 h. Two-way ANOVA was used for statistical analysis, and Sidak’s multiple comparisons test was used for comparisons between the two groups. The number of samples with biological replicates is shown as dots in a bar graph. *p* values are denoted with asterisks as follows: not significant (ns); ∗*p* value < 0.05; ∗∗*p* value < 0.01; ∗∗∗*p* value < 0.001; and ∗∗∗∗*p* value < 0.0001. (H and I) Representative flow cytometry pseudocolor map (H) and grouped histogram (I) showing the percentage of intracellular granzyme B-positive CAR-T cells at an E:T ratio of 1:1 after coculture for 24 and 72 h. Two-way ANOVA was used for statistical analysis, and Sidak’s multiple comparisons test was used for comparisons between the two groups. The number of samples with biological replicates is shown as dots in a bar graph. *p* values are denoted with asterisks as follows: not significant (ns); ∗*p* value < 0.05; ∗∗*p* value < 0.01; ∗∗∗*p* value < 0.001; and ∗∗∗∗*p* value < 0.0001. (J and K) Representative flow cytometry pseudocolor map (J) and grouped histogram (K) showing the percentage of intracellular IFN-γ and TNF-α-positive CAR-T cells at an E:T ratio of 1:1 after coculture for 24 and 72 h. Two-way ANOVA was used for statistical analysis, and Sidak’s multiple comparisons test was used for comparisons between the two groups. The number of samples with biological replicates is shown as dots in a bar graph. *p* values are denoted with asterisks as follows: not significant (ns); ∗*p* value < 0.05; ∗∗*p* value < 0.01; ∗∗∗*p* value < 0.001; and ∗∗∗∗*p* value < 0.0001.

To investigate the effects of asparagine metabolism on ALL cells and the killing efficacy of CAR-T cells, we designed an optimized coculture system involving ASNS overexpression (OE) NALM6 cancer cells and anti-CD19 CAR-T cells (Figure 1A). ASNS-OE NALM6-GL cancer cells containing ASNS cDNA, a luciferase reporter gene, and green fluorescent protein (GL) were generated and selected through lentivirus transduction and single-cell cloning. The protein expression of ASNS was confirmed by immunoblotting (Figures 1B and S1). Anti-CD19 CAR-T cells were produced through lentivirus spin infection, as described in previous studies.^15^^,^^17^^,^^18^ The synthetic CAR vector included a CD19 scFv (clone FMC63), a 4-1BB costimulatory domain, and a CD3ζ activation domain. Following lentivirus spin-infection of CD8-positive T cells for more than 14 days, high-purity CAR-T cells were enriched by flow cytometry (Figure 1C). CAR-T cells were cocultured with NALM6 cancer cells in parallel at the designated effector-to-target (E:T) ratio. The percentage of lysed NALM6 cells reflected the killing efficacy of CAR-T cells at 24 h (Figure 1D). However, ASNS-OE attenuated CAR-T-cell-mediated cancer cell lysis at E:T ratios of 1:1, 0.5:1, and 0.25:1 after 24 h of coculture. Even at an E:T ratio of 0.1:1 after 72 h of coculture, the percentage of lysed NALM6-GL cells was greater than that of ASNS-OE NALM6-GL cells (Figure 1E). The profiling of resident CAR-T cells and cancer cells was demonstrated by flow cytometry analysis after 72 h of coculture at E:T ratios of 1:1, 0.5:1, 0.25:1, and 0.1:1 (Figure 1F). The survival rate of CAR-T cells targeting NALM6-GL was higher than that of targeting ASNS-OE NALM6-GL cells at all four E:T ratios (Figure 1G). Consistently, the survival of NALM6-GL cells was lower in ASNS-OE NALM6-GL cell group than that in the NALM6-GL cell group at all four E:T ratios. Intracellular granzyme B-mediated target cell lysis also exhibited distinct profiles at a 1:1 E:T ratio (Figure 1H). There were significantly more granzyme B-positive CAR-T cells in the group targeting NALM6-GL cells than those targeting ASNS-OE NALM6-GL cells at both 24 and 72 h (Figure 1I). Similarly, distinct profiles of intracellular IFN-γ and TNF-α mediated target cell lysis were detected at a 1:1 E:T ratio (Figure 1J). There were significantly more IFN-γ and TNF-α positive CAR-T cells targeting NALM6-GL cells than those targeting ASNS-OE NALM6-GL at both 24 and 72 h (Figure 1K). These results confirmed that ASNS-OE in NALM6-GL cells attenuates CAR-T cell-mediated cancer cell killing efficacy.

### ASPG-OE CAR-T cells exerted superior killing effects on ASNS-OE cancer cells

**Figure 2:**
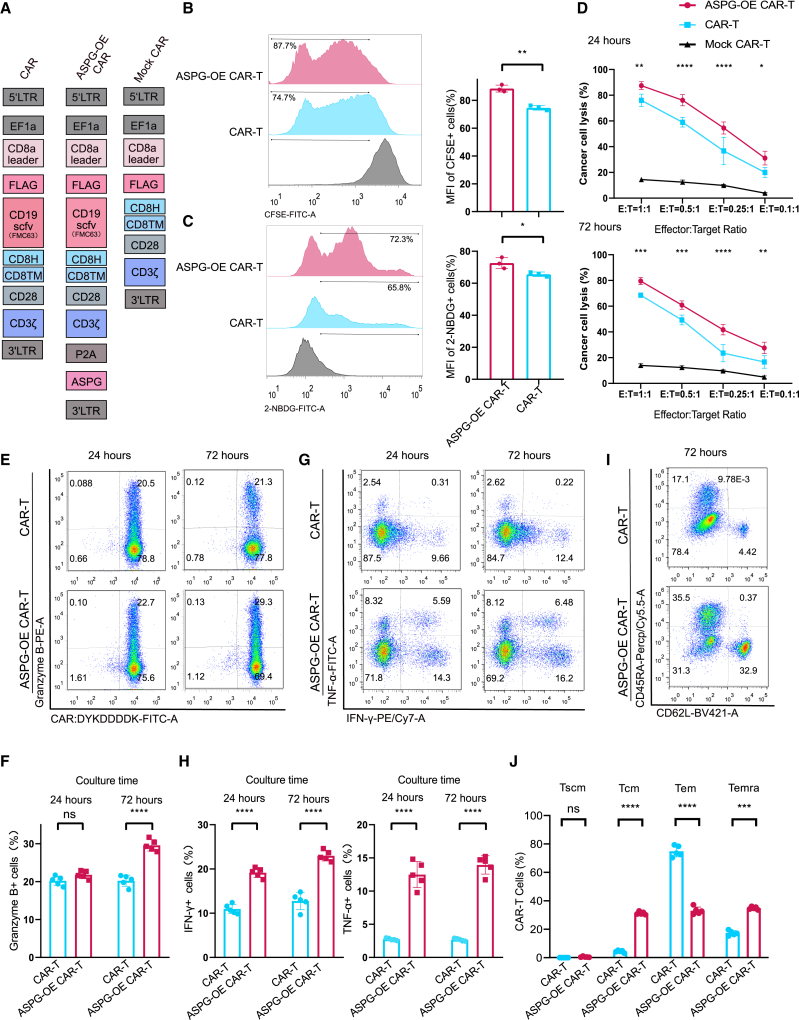
ASPG-OE CAR-T cells exerted superior killing efficiency against ASNS overexpression cancer cells (A) Schematic diagram showing the constructs of the CAR, the ASPG-OE CAR, and the mock CAR. (B) The typical flow cytometry stacked diagram and histogram showed a CFSE-based cell proliferation assay, revealing the distinct expansion capacity of ASPG-OE CAR-T cells. The *y* axis was labeled as mean fluorescence intensity (MFI) of CFSE+ cells. A two-sample t test was used for statistics. The number of samples with biological replicates is shown as dots in a bar graph. *p* values are denoted with asterisks as follows: not significant (ns); ∗*p* value < 0.05; ∗∗*p* value < 0.01; ∗∗∗*p* value < 0.001; and ∗∗∗∗*p* value < 0.0001. (C) The typical flow cytometry stacked diagram and histogram showed a 2-NBDG-based glucose uptake assay, revealing the distinct metabolic adaptation of ASPG-OE CAR-T cells. A two-sample t test was used for statistics. The number of samples with biological replicates is shown as dots in a bar graph. *p* values are denoted with asterisks as follows: not significant (ns); ∗*p* value < 0.05; ∗∗*p* value < 0.01; ∗∗∗*p* value < 0.001; and ∗∗∗∗*p* value < 0.0001. (D) The cancer cell lysis assay of effector ASPG-OE CAR-T cells to target ASNS-OE NALM6-GL cancer cells (E:T) at ratios of 1:1, 0.5:1, 0.25:1, and 0.1:1 after coculture for 24 h (up) and 72 h (down). Two-way ANOVA was used for statistical analysis, and Sidak’s multiple comparisons test was used for comparisons between the two groups. *p* values are denoted with asterisks as follows: not significant (ns); ∗*p* value < 0.05; ∗∗*p* value < 0.01; ∗∗∗*p* value < 0.001; and ∗∗∗∗*p* value < 0.0001. (E and F) The typical flow cytometry pseudocolor map (E) and grouped histogram (F) showed the intracellular granzyme B-positive ASPG-OE CAR-T cells and control CAR-T cells percentage at an E:T ratio of 1:1 after coculture for 24 and 72 h. Two-way ANOVA was used for statistical analysis, and Sidak’s multiple comparisons test was used for comparisons between the two groups. The number of samples with biological replicates is shown as dots in a bar graph. *p* values are denoted with asterisks as follows: not significant (ns); ∗*p* value < 0.05; ∗∗*p* value < 0.01; ∗∗∗*p* value < 0.001; and ∗∗∗∗*p* value < 0.0001. (G and H) The typical flow cytometry pseudocolor map (G) and grouped histogram (H) showed the intracellular IFN-γ- and TNF-α-positive ASPG-OE CAR-T cells and control CAR-T cells percentage at an E:T ratio of 1:1 after c-culture for 24 and 72 h. Two-way ANOVA was used for statistical analysis, and Sidak’s multiple comparisons test was used for comparisons between the two groups. The number of samples with biological replicates is shown as dots in a bar graph. *p* values are denoted with asterisks as follows: not significant (ns); ∗*p* value < 0.05; ∗∗*p* value < 0.01; ∗∗∗*p* value < 0.001; and ∗∗∗∗*p* value < 0.0001. (I and J) The typical flow cytometry pseudocolor map (I) and grouped histogram (J) showed the memory phenotype in the CAR^+^ CD8^+^ T cells of ASPG-OE CAR-T cells and control CAR-T cells at an E:T ratio of 1:1 after coculture for 72 h. The Tscm (CD45RA^+^ CD62L^+^ CD8^+^ CAR^+^), Tcm (CD45RA^−^ CD62L^+^ CD8^+^ CAR^+^), Tem (CD45RA^−^ CD62L^−^ CD8^+^ CAR^+^), and Temra (CD45RA^+^ CD62L^−^ CD8^+^ CAR^+^) were shown as percentages. Two-way ANOVA was used for statistical analysis, and Sidak’s multiple comparisons test was used for comparisons between the two groups. The number of samples with biological replicates is shown as dots in a bar graph. *p* values are denoted with asterisks as follows: not significant (ns); ∗*p* value < 0.05; ∗∗*p* value < 0.01; ∗∗∗*p* value < 0.001; and ∗∗∗∗*p* value < 0.0001.

To increase the anti-CD19 CAR-T cells mediated killing of ASNS-OE cancer cells, we engineered a CAR vector with *ASPG* (Figure 2A). A mock CAR-T cell vector lacking CD19 scFv (clone FMC63) served as the control. The successful generation of ASPG-OE CAR-T cells was confirmed by elevated *ASPG* mRNA and CAR expression in the responsible CAR-T cells (Figure S2). Proliferation assays demonstrated that the ASPG-OE CAR-T cells exhibited accelerated proliferation (Figure 2B). Metabolic adaptation was assessed using 2-NBDG uptake assays.^25^ The dynamic glucose uptake by ASPG-OE CAR-T cells indicated robust metabolic activity and adaptability (Figure 2C). To reveal the effective killing potential of ASPG-modified CAR-T cells, we designed a parallel *in vitro* killing assay in the coculture of ASPG-modified CAR-T cells with ASNS-OE NALM6 cancer cells (Figure 2D). When compared with mock CAR-T cells, CAR-T cells showed highly effective killing potential at 24 and 72 h. Furthermore, ASPG-OE CAR T cells showed enhanced cancer cell killing efficacy at all four E:T ratios. Intracellular granzyme B staining revealed a greater proportion of granzyme B-positive ASPG-OE CAR-T cells at a 1:1 E:T ratio after 24 h and 72 h of coculture (Figures 2E and 2F). Similarly, intracellular IFN-γ and TNF-α staining revealed increased proportions of IFN-γ- and TNF-α-positive cells in the ASPG-OE CAR-T cell group after 24 h and 72 h of coculture, respectively (Figures 2G and 2H). Given the association between metabolic reprogramming and distinct memory cell phenotypes in T cells,^26^ we investigated the expression of CD62L and CD45RA after 72 h of coculture at an E:T ratio of 1:1 using a CAR and CD45RA gating strategy (Figure 2I). Our findings were consistent with those of previous studies,^27^ revealing that after 72 h of coculture at an E:T ratio of 1:1, 4.37% of CAR-T cells exhibited central memory (Tcm, CD45RA^−^ CD62L^−^ CD8^+^ CAR^+^), 74.86% exhibited effector memory (Tem, CD45RA^−^ CD62L^−^ CD8^+^ CAR^+^), and 17.34% exhibited effector memory cells re-expressing CD45RA (Temra, CD45RA^+^ CD62L^−^ CD8^+^ CAR^+^) phenotypes (Figure 2J). Upon antigen exposure, CAR-T cells are activated and proliferate, resulting in progeny with effector and memory fates capable of providing immediate and long-term protection.^28^ The present study confirmed that ASPG-OE CAR-T cells underwent distinct memory differentiation since there were 31.32% of Tcm cells, 33.02% of Tem cells, and 34.82% of Temra cells in the ASPG-OE CAR-T cells after coculturing with ASNS-OE NALM6-GL cells. These results indicate that ASPG-OE CAR-T cells exhibit distinct differentiated phenotypic and functional features, contributing to their superior ability to kill cancer cells.

### ASPG-KO inhibited CAR-T-cell-mediated killing of ASNS-OE NALM6-GL cancer cells

**Figure 3:**
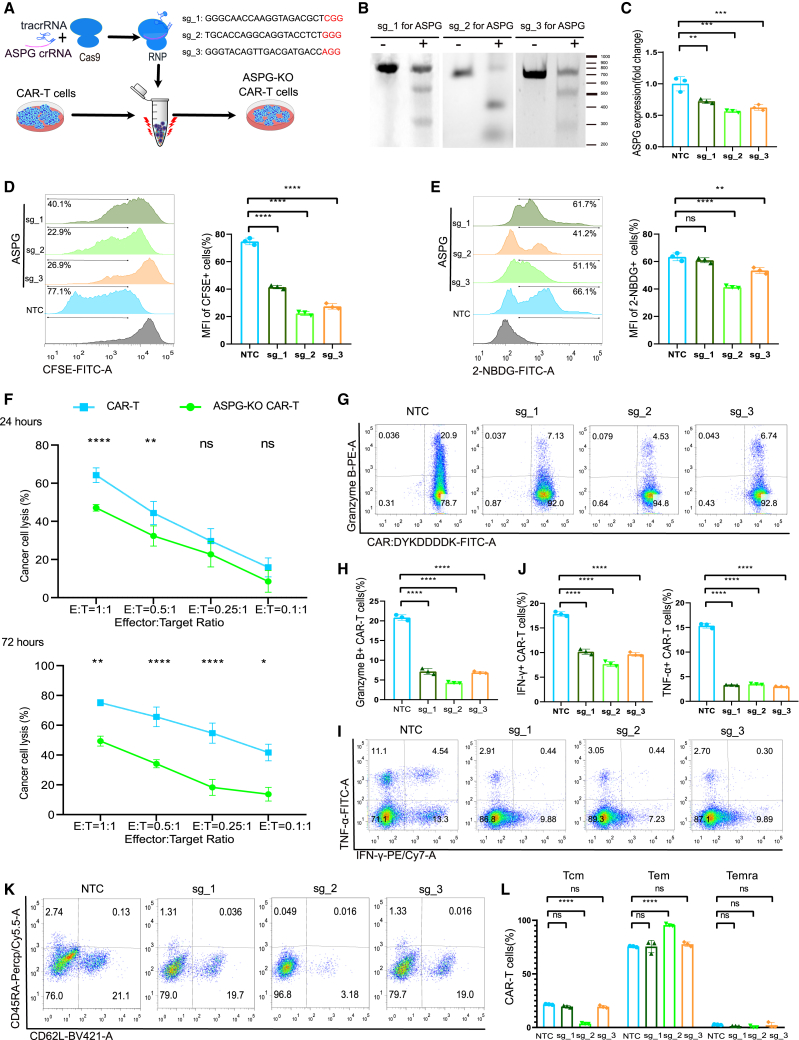
ASPG-KO abated CAR-T cell-mediated killing efficiency against ASNS-OE NALM6-GL cancer cells (A) Schematic diagram showing the knockout of ASPG from the CAR-T cells using RNP-mediated CRISPR-Cas9 method. The three sgRNA and protospacer adjacent motif sequences are shown. (B) The typical T7E1 mismatch detection assay showed that the *ASPG* gene was knocked out from three groups of ASPG-KO CAR-T cells. (C) The mRNA expression of ASPG in the three groups of ASPG-KO CAR-T cells was analyzed by relative quantitative PCR. One-way ANOVA was used for statistical analysis, and Sidak’s multiple comparisons test was used for comparison between the two groups. The number of samples with biological replicates is shown as dots in a bar graph. *p* values are denoted with asterisks as follows: not significant (ns); ∗*p* value < 0.05; ∗∗*p* value < 0.01; ∗∗∗*p* value < 0.001; and ∗∗∗∗*p* value < 0.0001. (D) The typical flow cytometry stacked diagram and histogram showed a CFSE-based cell proliferation assay, revealing the distinct expansion capacity of the three groups of ASPG-KO CAR-T cells. The *y* axis was labeled as MFI of CFSE+ cells. One-way ANOVA was used for statistical analysis, and Sidak’s multiple comparisons test was used for comparison between the two groups. The number of samples with biological replicates is shown as dots in a bar graph. *p* values are denoted with asterisks as follows: not significant (ns); ∗*p* value < 0.05; ∗∗*p* value < 0.01; ∗∗∗*p* value < 0.001; and ∗∗∗∗*p* value < 0.0001. (E) The typical flow cytometry stacked diagram and histogram showed a 2-NBDG-based glucose uptake assay, revealing distinct metabolic adaptation of the three groups of ASPG-KO CAR-T cells. The *y* axis was labeled as mean fluorescence intensity (MFI) of 2-NBDG+ cells. One-way ANOVA was used for statistical analysis, and Sidak’s multiple comparisons test was used for comparison between the two groups. The number of samples with biological replicates is shown as dots in a bar graph. *p* values are denoted with asterisks as follows: not significant (ns); ∗*p* value < 0.05; ∗∗*p* value < 0.01; ∗∗∗*p* value < 0.001; and ∗∗∗∗*p* value < 0.0001. (F) The cancer cell lysis assay of effector sg_2 of ASPG-KO CAR-T cells to target ASNS-OE NALM6-GL cancer cells (E:T) at a ratio of 1:1, 0.5:1, 0.25:1, and 0.1:1 from coculture for 24 h (up) and 72 h (down). Two-way ANOVA was used for statistical analysis, and Sidak’s multiple comparisons test was used for comparison between the two groups. *p* values are denoted with asterisks as follows: not significant (ns); ∗*p* value < 0.05; ∗∗*p* value < 0.01; ∗∗∗*p* value < 0.001; ∗∗∗∗*p* value < 0.0001. (G and H) The typical flow cytometry pseudocolor map (G) and histogram (H) showed the intracellular granzyme B-positive percentages from the three groups of ASPG-KO CAR-T cells at an E:T ratio of 1:1 after coculture for 72 h. One-way ANOVA was used for statistical analysis, and Sidak’s multiple comparisons test was used for comparison between the two groups. The number of samples with biological replicates is shown as dots in a bar graph. *p* values are denoted with asterisks as follows: not significant (ns); ∗*p* value < 0.05; ∗∗*p* value < 0.01; ∗∗∗*p* value < 0.001; and ∗∗∗∗*p* value < 0.0001. (I and J) The typical flow cytometry pseudocolor map (I) and histogram (J) showed the intracellular IFN-γ- and TNF-ɑ-positive percentages from the three groups of ASPG-KO CAR-T cells at an E:T ratio of 1:1 after coculture for 72 h. One-way ANOVA was used for statistical analysis, and Sidak’s multiple comparisons test was used for comparison between the two groups. The number of samples with biological replicates is shown as dots in a bar graph. *p* values are denoted with asterisks as follows: not significant (ns); ∗*p* value < 0.05; ∗∗*p* value < 0.01; ∗∗∗*p* value < 0.001; and ∗∗∗∗*p* value < 0.0001. (K and L) The typical flow cytometry pseudocolor map (K) and grouped histogram (L) showed the memory phenotype in the CAR^+^ CD8^+^ T cells percentages from the three groups of ASPG-KO CAR-T cells at an E:T ratio of 1:1 after coculture for 72 h. The Tcm (CD45RA^−^ CD62L^+^ CD8^+^ CAR^+^), Tem (CD45RA^−^ CD62L^−^ CD8^+^ CAR^+^), and Temra (CD45RA^+^ CD62L^−^ CD8^+^ CAR^+^) were shown as percentages. Two-way ANOVA was used for statistical analysis, and Sidak’s multiple comparisons test was used for comparison between the two groups. The number of samples with biological replicates is shown as dots in a bar graph. *p* values are denoted with asterisks as follows: not significant (ns); ∗*p* value < 0.05; ∗∗*p* value < 0.01; ∗∗∗*p* value < 0.001; and ∗∗∗∗*p* value < 0.0001.

To investigate the impact of ASPG-modified CAR-T cells on cancer cells, we used CRISPR-Cas9 to knock out ASPG in anti-CD19 CAR-T cells. We designed three single-guide RNAs (sgRNAs) targeting ASPG using CRISPick and selected them based on their on-target efficacy scores (Figure 3A). Cas9-sgRNA ribonucleoprotein (RNP) was delivered to anti-CD19 CAR-T cells via electroporation. All three sgRNAs showed a high knockout (KO) efficiency in the T7 endonuclease 1 (T7E1) mismatch detection assay (Figure 3B). The analysis confirmed that the three sgRNAs downregulated ASPG mRNA expression (27.7%–43.7%) (Figure 3C). Proliferation assays revealed that ASPG-KO CAR-T cells exhibited decreasing cell proliferation (Figure 3D). A metabolic adaptation assay using N-(7-Nitrobenz-2-oxa-1,3-diazol-4-yl)Amino)-2-Deoxyglucose (2-NBDG) uptake showed weaker glucose uptake in the sg_2 and sg_3 groups of the ASPG-KO CAR-T cells (Figure 3E). Although the three sgRNAs showed different degrees of KO efficiency, proliferation ability, and metabolic adaptation, the sg_2 group exhibited the lowest cancer cell killing activity in the killing assay after coculture with ASNS-OE CAR-T cells for 24 h (Figure S3). The percentages of cancer cells lysed by ASPG-KO CAR-T cells from the sg_2 group were measured after coculturing for 24 and 72 h (Figure 3F). ASPG-KO CAR-T cells showed a significant decrease in cancer cell killing efficacy, especially at the effector cell ratios of 1:4 and 1:10. Intracellular granzyme B staining revealed fewer granzyme B-positive cells in all three sgRNA groups of ASPG-KO CAR-T cells at an E:T ratio of 1:1 after coculture for 72 h (Figures 3G and 3H). The results of intracellular IFN-γ and TNF-α staining also revealed decreased numbers of IFN-γ- and TNF-α-positive cells in the three sgRNA groups of ASPG-KO CAR-T cells after coculture for 72 h (Figures 3I and 3J). The distinct memory cell phenotype of CAR-T cells was assessed based on the expression of CD62L and CD45RA.^14^^,^^27^ The majority of ASPG-KO CAR-T cells in the sg_2 group exhibited a Tem phenotype, whereas those in the sg_1 and sg_3 groups exhibited the memory phenotype similar to that of the control CAR-T cells after coculturing for 72 h at an E:T ratio of 1:1 (Figure 3K). An overwhelming increase in the number of Tems of ASPG-KO CAR-T cells demonstrated reduced killing of ASNS-OE cancer cells (Figure 3L). Based on the comparison of sgRNA-mediated KO efficiency, proliferation ability, metabolic adaptation, and cancer cell killing efficacy, we selected sg_2 for subsequent ASPG-KO experiments with nucleotide sequence as 5′-TGCACCAGGCAGGTACCTCT-3'.

### ASPG-OE and ASPG-KO CAR-T cells showed opposite anticancer effects on ASNS-OE NALM6-GL cancer cells

**Figure 4:**
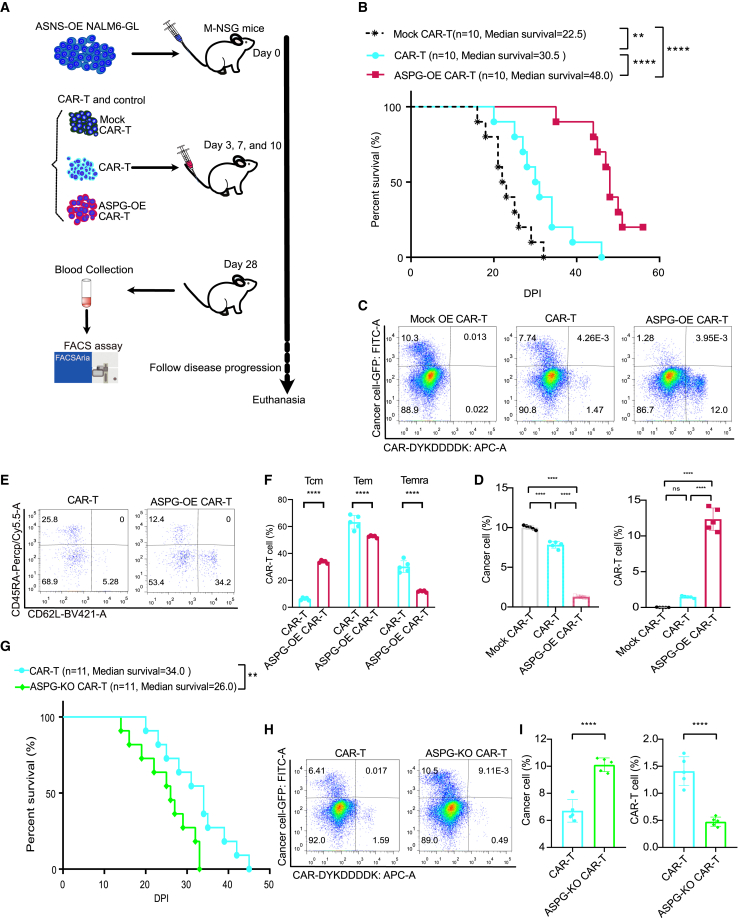
ASPG-OE and ASPG-KO CAR-T cells showed opposite anticancer activity against ASNS-OE NALM6-GL cancer cells (A) Schematic diagram of the *in vivo* xenogenic mouse model of ASPG-modified CAR-T cells against ASNS-OE NALM6-GL cancer. (B) Overall survival of mice bearing ASNS-OE NALM6-GL cancer after receiving CAR-T and ASPG-OE CAR-T cell therapy. Survival data were analyzed using the Kaplan-Meier method, and survival probabilities among groups were compared using a log rank test. *p* values are denoted with asterisks as follows: not significant (ns); ∗*p* value < 0.05; ∗∗*p* value < 0.01; ∗∗∗*p* value < 0.001; ∗∗∗∗*p* value < 0.0001. (C and D) Representative flow cytometry pseudocolor diagram (C) and histogram (D) showing resident ASNS-OE NALM6-GL cancer cells and CAR-T cells in the blood of mice at day 28 after receiving CAR-T and ASPG-OE CAR-T therapy. One-way ANOVA was used for statistical analysis, and Sidak’s multiple comparisons test was used for comparison between the two groups. The number of samples with biological replicates is shown as dots in a bar graph. *p* values are denoted with asterisks as follows: not significant (ns); ∗*p* value < 0.05; ∗∗*p* value < 0.01; ∗∗∗*p* value < 0.001; and ∗∗∗∗*p* value < 0.0001. (E and F) Representative flow cytometry pseudocolor map (E) and grouped histogram (F) showing the memory phenotype of CAR-T cells in the blood of mice at day 28 after receiving CAR-T and ASPG-OE CAR-T therapy. Tcm (CD45RA^−^ CD62L^+^ CD8^+^ CAR^+^), Tem (CD45RA^−^ CD62L^−^ CD8^+^ CAR^+^), and Temra (CD45RA^+^ CD62L^−^ CD8^+^ CAR^+^) are shown as percentages. Two-way ANOVA was used for statistical analysis, and Sidak’s multiple comparisons test was used for comparison between the two groups. The number of samples with biological replicates is shown as dots in a bar graph. *p* values are denoted with asterisks as follows: not significant (ns); ∗*p* value < 0.05; ∗∗*p* value < 0.01; ∗∗∗*p* value < 0.001; and ∗∗∗∗*p* value < 0.0001. (G) Overall survival of mice bearing ASNS-OE NALM6-GL cancer after receiving CAR-T and SPG-KO CAR-T cell therapy. Survival data were analyzed using the Kaplan-Meier method, and survival probabilities among groups were compared using a log rank test. *p* values are denoted with asterisks as follows: not significant (ns); ∗*p* value < 0.05; ∗∗*p* value < 0.01; ∗∗∗*p* value < 0.001; ∗∗∗∗ and *p* value < 0.0001. (H and I) Representative flow cytometry pseudocolor diagram (H) and histogram (I) showing resident ASNS-OE NALM6-GL cancer cells and CAR-T cells in the blood of mice at day 28 after receiving CAR-T and ASPG-KO CAR-T therapy. One-way ANOVA was used for statistical analysis, and Sidak’s multiple comparisons test was used for comparison between the two groups. The number of samples with biological replicates is shown as dots in a bar graph. *p* values are denoted with asterisks as follows: not significant (ns); ∗*p* value < 0.05; ∗∗*p* value < 0.01; ∗∗∗*p* value < 0.001; and ∗∗∗∗*p* value < 0.0001.

The *in vivo* anticancer experiment was adapted from previous studies.^15^^,^^18^ Specifically, 1 million NALM6-GL cancer cells were injected into the tail vein on day 0 (Figure 4A). ASPG-modified CAR-T cells (2 million) were administered three times on days 3, 7, and 10 post inoculation, along with 1 μg of recombinant humanized interleukin-2 (IL-2) per mouse every other day beginning on day 2.^29^ Compared to mice treated with mock CAR-T cells, mice treated with modified CAR-T cells exhibited prolonged survival (the median survival time increased from 22.5 to 30.5 days). Notably, the ASPG-OE CAR-T cells extended the overall survival to 48.0 days (Figure 4B). Blood collected on day 28 was analyzed for resident cancer cells and CAR-T cell composition (Figure 4C). There were no CAR-T cells in the mock CAR-T cells treated group, while 10.3% of the cells were resident cancer cells. The CAR-T cell treatment groups showed 7.74% in the number of resident cancer cells and 1.47% in the number of resident CAR-T cells in the blood. In contrast, ASPG-OE CAR-T-cell treatment decreased the percentages of residual cancer cells to 1.28% and increased the percentage of residual CAR-T cells to 12.0% on day 28. These results indicated the superior anticancer activity and viability of ASPG-OE CAR-T cells (Figure 4D). The memory cell phenotype of CAR-T cells was characterized by the expression of CD62L, CD45RA, CD44, CAR, and CD8 (Figures 4E and S4). ASPG-OE CAR-T cells exhibited an increase of Tcm (33.80%) when compared to control CAR-T cells (6.21%), with a substantial decrease of Tem (52.58%) to control CAR-T cells (63.20%, and Temra (11.84%) to control CAR-T cells (30.24%) (Figure 4F). Subsequently, the anticancer effect mediated by ASPG-KO CAR-T cells was investigated in a second set of experiments (Figure 4G). Compared with CAR-T cell therapy, ASPG-KO CAR-T cell therapy resulted in poorer survival (median survival decreased from 34.0 days to 26.0 days). Consistent with previous results, 6.41% resident cancer cells and 1.59% CAR-T cells were observed in the CAR-T-cell treatment group. Resident cancer cells (10.5%) were evident in the blood of the ASPG-KO CAR-T cell group on day 28 (Figure 4H). The substantial increase in cancer cells and decrease in CAR-T cells in the ASPG-KO CAR-T-cell group indicated their inferior anticancer activity and survivability (Figure 4I). Taken together, these results suggest that ASPG-OE CAR-T cells exhibit superior viability and anticancer activity against ASNS-OE cancer cells, whereas ASPG-KO CAR-T cells exhibit inferior viability and anticancer activity.

### ASPG-OE CAR-T cells showed increased anticancer activity against NALM6-GL cancer cells

**Figure 5:**
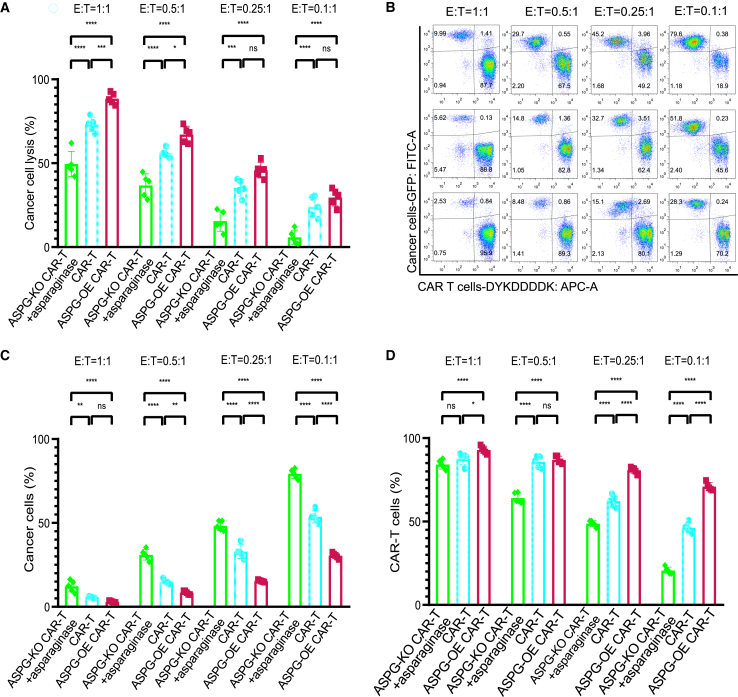
ASPG-OE CAR-T cells exhibited enhanced anticancer activity against ASNS-OE NALM6-GL cancer cells (A) Grouped histogram showing the cancer cell lysis assay of effector ASPG-modified CAR-T cells against ASNS-OE NALM6-GL cancer cells (E:T) at ratios of 1:1, 0.5:1, 0.25:1, and 0.1:1 after coculture for 24 h. Two-way ANOVA was used for statistical analysis, and Sidak’s multiple comparisons test was used for comparisons between the two groups. The number of samples with biological replicates is shown as dots in a bar graph. *p* values are denoted with asterisks as follows: not significant (ns); ∗*p* value < 0.05; ∗∗*p* value < 0.01; ∗∗∗*p* value < 0.001; and ∗∗∗∗*p* value < 0.0001. (B–D) Representative flow cytometry pseudocolor diagram (B) and grouped histogram showing the percentage of residual ASNS-OE NALM6-GL cells (C) and the percentage of CAR-T cells responsible for killing (D) at an E:T ratio of 1:1 after coculture for 24 h. Two-way ANOVA was used for statistical analysis, and Sidak’s multiple comparisons test was used for comparisons between the two groups. The number of samples with biological replicates is shown as dots in a bar graph. *p* values are denoted with asterisks as follows: not significant (ns); ∗*p* value < 0.05; ∗∗*p* value < 0.01; ∗∗∗*p* value < 0.001; and ∗∗∗∗*p* value < 0.0001.

The ASNS protein has been reported to be expressed in lymphoid blasts, suggesting its role in cancer progression, drug resistance, and immunotherapy.^30^^,^^31^ Moreover, ASNS overexpression in leukemia cells is associated with resistance to immunotherapy.^32^ ASNS-OE NALM6-GL cancer cells also exhibited resistance to CAR-T-cell-mediated cell lysis. Interestingly, ASPG-OE CAR-T cells exhibited a distinct memory T cell phenotype with increased numbers of Tcm cells and decreased Tem and Temra cells. This memory T cell phenotype has been reported to be related to therapeutic efficacy and persistence.^33^ Therefore, we compared the cancer cell lysis abilities of ASPG-OE CAR-T cells and ASPG-KO CAR-T cells cotreated with ASPG. CAR-T cells exhibited a gradual decrease in cancer cell lysis efficacy when the CAR-T cell ratio was gradually reduced from 1:1 to 0.1:1 after coculture for 24 h (Figure 5A). ASPG-OE CAR-T cells demonstrated superior anticancer activity against NALM6-GL cancer cells at E:T ratios of 1:1, 0.5:1, and 0.25:1. This finding was consistent with previous results showing that ASPG-OE CAR-T cells exhibited superior viability and anticancer activity. However, even with the addition of 0.5 IU/mL ASPG,^34^ ASPG-KO CAR-T cells showed significantly decreasing cancer lysis efficacy at E:T ratios of 1:1, 0.5:1, 0.25:1, and 0.1:1. Moreover, resident CAR-T cells and cancer cells were analyzed by flow cytometry after coculture (Figure 5B). The opposite trend was observed for the ASPG-KO and ASPG-OE CAR-T cells: the ASPG-KO CAR-T cells exhibited inferior survival and cancer cell lysis efficacy, whereas the ASPG-OE CAR-T cells demonstrated superior survival and cancer cell lysis efficacy (Figures 5C and 5D). The significantly decreasing cancer lysis efficacy of ASPG-KO CAR-T cells could not be rescued by cotreatment with ASPG, indicating phenotypic and functional reprogramming after ASPG modification.

### ASPG-modified CAR-T cells showed distinct phenotypic and functional features

**Figure 6:**
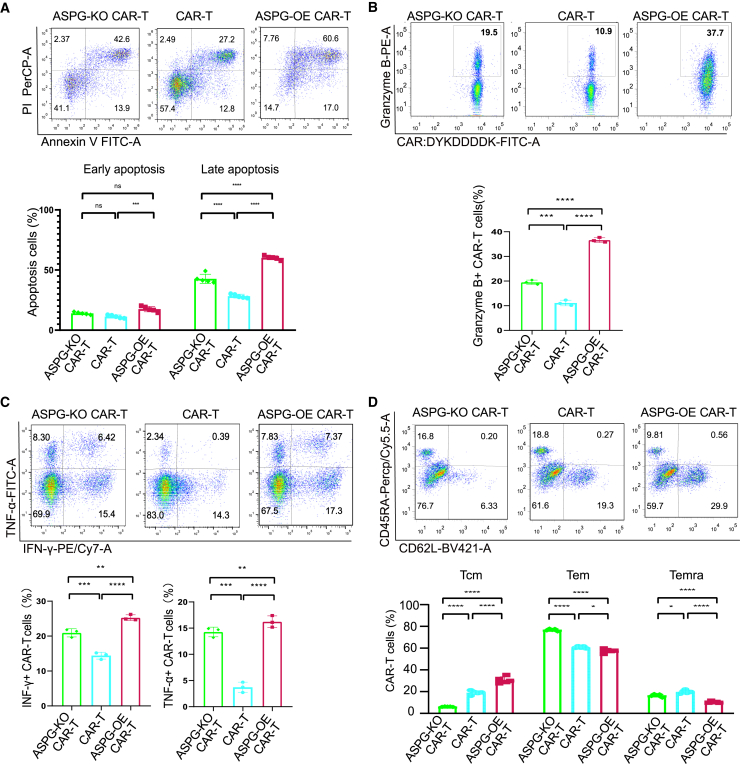
ASPG-modified CAR-T cells showed distinct phenotypic and functional features (A) The typical flow cytometry pseudocolor map and grouped histogram show the apoptosis percentages of NALM6-GL cancer cells from the ASPG-modified CAR-T cell treatment at an E:T ratio of 1:1 after coculture for 24 h. Two-way ANOVA was used for statistical analysis, and Sidak’s multiple comparisons test was used for comparison between the two groups. The number of samples with biological replicates is shown as a dot in the bar graph. *p* values are denoted with asterisks as follows: not significant (ns), ∗*p* value < 0.05; ∗∗*p* value < 0.01; ∗∗∗*p* value < 0.001; and ∗∗∗∗*p* value < 0.0001. (B) The typical flow cytometry pseudocolor map and histogram showed intracellular granzyme B-positive percentages. One-way ANOVA was used for statistical analysis, and Sidak’s multiple comparisons test was used for comparison between the two groups. The number of samples with biological replicates is shown as a dot in the bar graph. *p* values are denoted with asterisks as follows: not significant (ns), ∗*p* value < 0.05; ∗∗*p* value < 0.01; ∗∗∗*p* value < 0.001; and ∗∗∗∗*p* value < 0.0001. (C) Typical flow cytometry pseudocolor map and histogram showing the intracellular IFN-γ- and TNF-ɑ-positive percentages from the ASPG-modified CAR-T cells. One-way ANOVA was used for statistical analysis, and Sidak’s multiple comparisons test was used for comparison between the two groups. The number of samples with biological replicates is shown as a dot in the bar graph. *p* values are denoted with asterisks as follows: not significant (ns), ∗*p* value < 0.05; ∗∗*p* value < 0.01; ∗∗∗*p* value < 0.001; and ∗∗∗∗*p* value < 0.0001. (D) Typical flow cytometry pseudocolor map and grouped histogram showing the memory phenotype in CAR^+^ CD8^+^ T cells from the ASPG-modified CAR-T cells after culture for 14 days. Tcm (CD45RA^−^ CD62L^+^), Tem (CD45RA^−^ CD62L^−^), and Temra (CD45RA^+^ CD62L^−^) are shown as percentages. Two-way ANOVA was used for statistical analysis, and Sidak’s multiple comparisons test was used for comparisons between the two groups. The number of samples with biological replicates is shown as a dot in the bar graph. *p* values are denoted with asterisks as follows: not significant (ns), ∗*p* value < 0.05; ∗∗*p* value < 0.01; ∗∗∗*p* value < 0.001; and ∗∗∗∗*p* value < 0.0001.

The functional and phenotypic characteristics of ASPG-OE and ASPG-KO CAR-T cells were compared in parallel.^15^^,^^17^^,^^18^ First, the apoptosis assay demonstrated that ASPG-OE CAR-T cells produced a greater percentage of apoptotic cells than control CAR-T cells after coculture for 24 h. In contrast, ASPG-KO CAR-T cells produced a decreasing percentage of apoptotic cells, even after the addition of ASPG for 24 h (Figure 6A). After coculture for 72 h at an E:T ratio of 1:1, the resident ASPG-modified CAR-T cells were sorted and expanded for phenotypic analysis, and the cancer cells were removed. The results of intracellular granzyme B staining revealed that ASPG-OE CAR-T cells generated a greater number of granzyme B-positive cells (Figure 6B). Additionally, the results of intracellular IFN-γ and TNF-α staining also revealed increased production of IFN-γ- and TNF-α-positive cells in the ASPG-OE CAR-T cell group (Figure 6C). The composition of ASPG-OE CAR-T cells was 30.64% Tcms, 57.50% Tems, and 10.39% Temra cells, while the composition of ASPG-KO CAR-T cells was 6.14% Tcms, 76.78% Tems, and 16.48% Temra cells (Figure 6D). ASPG-modified CAR-T cells showed distinct memory differentiation as ASPG-OE CAR-T cells displayed an increasing percentage Tcm and decreasing Tem and Temra. These results illustrate that ASPG expression in CAR-T cells leads to distinct phenotypic characteristics and cancer cell killing abilities. Specifically, ASPG-OE CAR-T cells exhibited superior cancer cell lysis efficacy, whereas ASPG-KO CAR-T cells demonstrated inferior cancer cell lysis efficacy.

### ASPG-OE CAR-T cells showed superior anticancer activity against NALM6-GL cancer cells, while ASPG-KO CAR-T cells showed inferior anticancer activity

**Figure 7:**
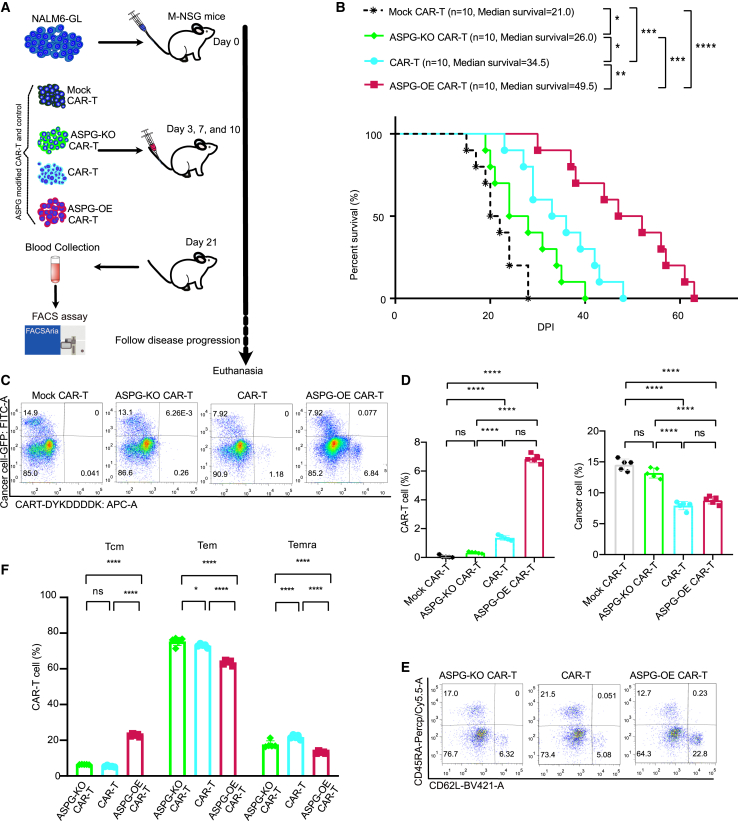
ASPG-OE CAR-T cells showed superior anticancer activity against NALM6-GL cancer cells, while ASPG-KO CAR-T cells showed inferior anticancer activity (A) Schematic diagram of the *in vivo* xenogenic mouse model of ASPG-modified CAR-T cells against NALM6-GL cancer. (B) Overall survival of mice bearing NALM6-GL cancer after receiving CAR-T and ASPG-modified CAR-T cell therapy. Survival data were analyzed using the Kaplan-Meier method, and survival probabilities among groups were compared using a log rank test. *p* values are denoted with asterisks as follows: not significant (ns); ∗*p* value < 0.05; ∗∗*p* value < 0.01; ∗∗∗*p* value < 0.001; and ∗∗∗∗*p* value < 0.0001. (C and D) Representative flow cytometry pseudocolor diagram (C) and histogram (D) showing resident NALM6-GL cancer cells and CAR-T cells in the blood of mice at day 21 after receiving CAR-T- and ASPG-modified CAR-T therapy. One-way ANOVA was used for statistical analysis, and Sidak’s multiple comparisons test was used for comparison between the two groups. The number of samples with biological replicates is shown as dots in a bar graph. *p* values are denoted with asterisks as follows: not significant (ns); ∗*p* value < 0.05; ∗∗*p* value < 0.01; ∗∗∗*p* value < 0.001; and ∗∗∗∗*p* value < 0.0001. (E and F) Representative flow cytometry pseudocolor map (E) and grouped histogram (F) showing the memory phenotype of CAR-T cells in the blood of mice at day 21 after receiving CAR-T and ASPG-modified CAR-T therapy. Tcm (CD45RA^−^ CD62L^+^), Tem (CD45RA^−^ CD62L^−^), and Temra (CD45RA^+^ CD62L^−^) are shown as percentages. Two-way ANOVA was used for statistical analysis, and Sidak’s multiple comparisons test was used for comparison between the two groups. The number of samples with biological replicates is shown as dots in a bar graph. *p* values are denoted with asterisks as follows: not significant (ns); ∗ *p* value < 0.05; ∗∗*p* value < 0.01; ∗∗∗*p* value < 0.001; and ∗∗∗∗*p* value < 0.0001.

An *in vivo* anticancer experiment involving ASPG-modified CAR-T cells targeting NALM6-GL cells was conducted using a previously established method (Figure 7A). One million NALM6-GL cancer cells were injected via the tail vein on day 0. Subsequently, 2 million ASPG-modified CAR-T cells were administered 3, 7, and 10 days post-inoculation through tail vein injection, along with the administration of 1 μg of recombinant humanized IL-2 per mouse every other day.^29^ Compared to mice treated with mock CAR-T cells, mice treated with ASPG-OE CAR-T cells exhibited prolonged survival (the median survival time increased from 21.0 days to 34.5 days). Specifically, ASPG-OE CAR-T cells extended the overall survival to 49.5 days, while ASPG-KO CAR-T-cell treatment reduced the survival to 26.0 days (Figure 7B). On day 21, the resident cancer cells and CAR-T cells were collected from the blood (Figure 7C). Resident cancer cells were found in all four groups, whereas different percentages of CAR-T cells were detected only in the CAR-T-cell treatment groups. Treatment with ASPG-OE CAR-T cells resulted in 6.87% resident CAR-T cells and 8.79% resident NALM6-GL cancer cells, whereas treatment with ASPG-KO CAR-T cells resulted in 0.34% resident CAR-T cells and 13.20% resident NALM6-GL cancer cells (Figure 7D). The substantial reduction in cancer cells and the preservation of resident CAR-T cells indicated the superior anticancer activity and viability of ASPG-OE CAR-T cells. The memory cell phenotype of the CAR-T cells was also characterized (Figure 7E). CAR-T cells exhibited a substantial proportion of Tem cells, as shown in previous research.^33^ However, ASPG-KO CAR-T cells exhibited a significant increase in the percentage of Tem cells and a decrease in Temra cells (Figure 7F). In contrast, ASPG-OE in CAR-T cells switched the memory differentiation features with a significant increase in Tcm cells and a decrease in Tem and Temras. These results confirm that ASPG-OE CAR-T cells possess superior anticancer potential by differentiating into Tcm cells, which leads to enhanced anticancer efficiency.

## Discussion

In this study, we explored the relationship between ASNS and the treatment of leukemia, as well as the usage of ASPG in ALL treatment. Initially, we observed that ASNS OE reduced the cancer cell killing efficacy of CAR-T cells. Subsequently, we generated ASPG-modified CAR-T cells and obtained consistent *in vitro* results indicating that ASPG-OE CAR-T cells exhibited enhanced killing of ASNS-OE cancer cells, whereas ASPG-KO attenuated the CAR-T cell-mediated cancer cell lysis effect on ASNS-OE NALM6-GL cancer cells. *In vivo* experiments confirmed that ASPG-OE CAR-T cells demonstrated superior viability and anticancer activity against ASNS-OE cancer cells, whereas ASPG-KO CAR-T cells exhibited inferior viability and decreased anticancer activity. Furthermore, considering the low ASNS protein expression in lymphoid blasts, we investigated the effect of ASPG-modified CAR-T cells on NALM6-GL cancer cells. Our findings revealed that the ASPG-OE CAR-T cells leveraged asparagine metabolic reprogramming to demonstrate superior anticancer activity and distinct phenotypic features in both *in vitro* coculture experiments and *in vivo* xenograft mouse models.

Asparagine is a nonessential amino acid that needs to be obtained from external sources because of its limited endogenous synthesis.^35^ This nonessential nature of asparagine is particularly relevant in certain types of hematologic malignancies with metabolic demands related to high levels of ASNS.^36^ ASNS is the key enzyme responsible for asparagine synthesis in ALL.^31^ Therefore, the availability of asparagine in the tumor microenvironment is crucial for the growth and treatment of ALL. Thus, ASPG was approved for ALL in adult and pediatric patients as an effective chemotherapeutic regimen. ASPG breaks down circulating asparagine in the blood, thereby depriving cancer cells of this nonessential amino acid and inhibiting their growth.^37^ This approach to asparagine depletion therapy has been found to be effective in reducing the levels of circulating asparagine and inhibiting the growth of cancer cells in some patients with leukemia. Clinically, ASPG has been used in combination with other immunotherapies, such as antibody therapy and CAR-T cell therapy.^38^ We thus coupled *ASPG* gene with CAR-T cell therapy for the treatment of ALL.

CAR-T cell-based immunotherapy has revolutionized the field of oncology, offering a highly innovative and effective approach for treating certain types of hematologic malignancies and potentially providing a curative treatment option.^39^ This therapy has demonstrated remarkable success in treating certain leukemias, leading to durable remission in patients. Technological advancements and clinical developments continue to drive the evolution of CAR-T-cell therapy toward a personalized medical approach for relapsed/refractory hematologic malignancies.^40^ A recent study revealed that asparagine restriction in CD8^+^ T cells can have opposing effects during the activation and differentiation states, enhancing metabolic fitness and antitumor functionality through an NFE2 Like BZIP Transcription Factor 2 (NRF2) - dependent stress response.^21^ Metabolic reprogramming is often observed in different states of T cell proliferation, activation, and exhaustion.^41^ CAR-dependent metabolic reprogramming has been shown to be associated with cellular energetics, nutrient utilization, proliferation, metabolic adaptation, and differentiation status.^42^ Our experiments demonstrated the distinct phenotypic states of ASPG-modified CAR-T cells. Upon coculture and antigen clearance, ASPG-OE CAR-T cells gradually progressed into Tcm cells, which mediate long-term protection against cancer cells.

ASPG plays a crucial role in the treatment of certain types of lymphoma and leukemia, particularly ALL^43^ and non-Hodgkin lymphomas.^44^ The dosage, duration, indications, and serum asparagine levels are crucial factors to consider to ensure safe and effective usage.^45^ Building on the mechanism of ASPG-mediated asparagine depletion in the treatment of hematologic malignancies, we developed ASPG-modified CAR-T cells for the treatment of ALL. However, there are two limitations that need to be addressed. First, hypersensitivity reactions of ASPG-OE CAR-T cells may lead to decreased ASPG activity. Careful monitoring of hypersensitivity reactions is important during treatment.^46^ Second, serum asparagine levels are crucial for ensuring effective asparagine depletion in the treatment of ALL.^45^ High levels of asparagine may be associated with a range of adverse effects, including hepatotoxicity, pancreatitis, coagulopathy, hyperglycemia, and allergic reactions.^47^ Monitoring serum asparagine levels and adjusting the replacement treatment is important for the ASPG-OE CAR-T cell therapy based on adequate disease control.^48^

In conclusion, this study demonstrated the importance of ASNS in immunotherapy resistance and the potential application of the enzyme ASPG in the treatment of hematologic malignancies. *In vitro* coculture experiments demonstrated that the ASPG-OE CAR-T cells exhibited increasing killing efficacy against ASNS-OE cancer cells, whereas ASPG-KO CAR-T cells showed decreasing killing efficiency. *In vivo* xenograft mouse experiments further confirmed the superior survivability and the anticancer activity of ASPG-OE CAR-T cells against ASNS-OE cancer cells, in contrast to the inferior viability and anticancer activity of ASPG-KO CAR-T cells. Functional analyses revealed that ASPG-OE CAR-T cells exploited asparagine metabolic reprogramming to phenotype switch into differentiating into Tcms and Temra cells, which provided long-term protection against cancer. The importance of ASNS in immunotherapy resistance supports the potential application of ASPG-modified CAR-T cells for further advances in the treatment of hematologic malignancies.

## Materials and methods

### Isolation and culture of primary human CD8^+^ T lymphocytes

Peripheral blood mononuclear cell samples were obtained from healthy donors with the approval of the Ethics Committee of Shanghai Sixth People’s Hospital Affiliated Shanghai Jiao Tong University School of Medicine (no. 2021-YS-0215) after obtaining written informed consent. Primary human CD8^+^ T cells were isolated using the MojoSort Human CD8 T cell Isolation Kit (BioLegend Technologies), resulting in a purity exceeding 95%. CD8^+^ T lymphocytes were then activated with anti-CD3 and anti-CD28 antibodies (Thermo Fisher Scientific Inc.) at a concentration of 1 μg/mL and cultured on RetroNectin (Takarabio) pre-coated plates. The T cells were expanded in medium consisting of 90% RPMI 1640 supplemented with 10% fetal bovine serum (FBS, Gibco, Thermo Fisher Scientific Inc.), 30 U/mL recombinant human IL-2 (PeproTech), 0.05 mM 2-mercaptoethanol (Sigma-Aldrich), and 100 IU/mL penicillin-streptomycin (HyClone). The starting concentration was 1 × 10^6^ cells/mL.

### Anti-CD19 CAR construction

The synthetic anti-CD19 CAR vector consisted of several components, including the CD19 scFv (FMC63 and MP121303.1), 4-1BB costimulatory domain (nucleotides 640–765; NM_001561.5), and CD3ζ activation domain (nucleotides 160–492; NM_198053.2). To express the anti-CD19 CAR, a second-generation lentiviral vector transfer plasmid regulated by the human HBS1 like translational GTPase (EF-1α) promoter was generated via overlapping PCR. Additionally, the vectors were equipped with a FLAG tag to quantify the transduction efficiency and CAR expression. Mock CARs with an anti-CD19 CAR and without a CD19 scFv were created. The ASPG-OE CAR construct was designed by integrating ASPG cDNA (nucleotides 72–1793; NM_001080464.3) after T2A into the anti-CD19 CAR vector via overlapping PCR. The oligos are included in Table S1.

### Lentivirus production

HEK293T cells were initially seeded at 8 × 10^6^ cells in a 100-mm dish one day before transduction. After 24 h, 80% confluent cells were prepared, and the lentivirus was generated by cotransfecting HEK293T cells with plasmids encoding anti-CD19 CAR moieties (16.5 μg), pMD.2G encoding the VSV-G envelope (7.5 μg), and the packaging vector psPAX2 (13.5 μg) using a polyethylenimine transfection protocol according to the manufacturer’s instructions (Sigma-Aldrich). After 48 h, the supernatant was collected and filtered through a 0.45-μm membrane to eliminate cell debris. The virus was concentrated using an Amicon Ultra15 regenerated cellulose membrane (Millipore, USA). Subsequently, the virus was suspended in RPMI 1640 media, divided into 100-μL aliquots, and stored at −80°C. The viral titer was determined by viral transduction of HEK293T cells, followed by the measurement of CAR expression.

### Anti-CD19 CAR-T-cell and ASPG-OE CAR-T-cell production

Activated CD8^+^ T lymphocytes were transduced with lentiviral vectors using RetroNectin (Takarabio) pre-coated plates supplemented with polybrene (TR-1003-G, Sigma) at a concentration of 8 μg/mL. The transduction was followed by centrifugation for 90 min at 350 × *g* and then incubation at 37°C. After 12 h, the virus supernatant was removed, and the T cells were expanded in the conditioned medium as previously described.^29^^,^^49^ The genetically modified CAR-T cells were maintained in a complete T cell medium supplemented with 10 ng/mL IL-2, which was replenished twice a week, and used for functional assays 14 days after transduction. ASPG-OE CAR-T cells were generated using the same protocol.

### ASPG-KO CAR-T-cell production

We generated ASPG-KO CAR-T cell lines using CRISPR-Cas9. gRNA was created by duplexing two single RNA molecules in a 1:1 ratio of CRISPR RNA (crRNA) to trans-activating CRISPR RNA (tracrRNA). The sgRNAs targeting ASPG were designed using CRISPick (https://portals.broadinstitute.org/gppx/crispick/public), and the crRNA and protospacer adjacent motif (PAM) sequences were selected based on maximum on-target efficacy scores as follows: sg_1, 5′-AAGGCAAACCCACTCAGCGA TGG-3′; sg_2, 5′-GGGCAACCAAGGTAGACGCT CGG-3′; and sg_3, 5′-GGGTACAGTTGACGATGACC AGG-3′. The designed crRNA and tracrRNA were obtained from GenScript (Nanjing, China). The RNP complex was assembled by combining gRNA with GenCRISPR-Cas9 nuclease (GenScript, Nanjing, China). Before nucleofection, the cells were pretreated with 20 μM DNA ligase IV inhibitor 2,3-dihydro-5,6-bis[(E)-(phenylmethylene)amino]-2-thioxo-4(1H)-pyrimidinone (SCR7) (Selleck, Shanghai, China) for 4 h to inhibit non-homologous end-joining and promote homology-directed repair. The 4D-Nucleofector Kit S (Lonza, Guangzhou, China) was used to transfect CAR-T cells with the RNP complex. The cells were then incubated at 37°C for 2 days, single-cell cloned by limited dilution, and expanded. The KO efficacy was determined using a mismatch detection assay with T7E1 (NEB, Shanghai, China).

### Construction of luciferase-expressing and ASNS-OE cancer cells

Wild-type NALM6 cells were obtained from Procell Life Science & Technology (Wuhan, China) and thawed at 37°C. The cells were cultured in complete RPMI-1640 medium supplemented with 10% FBS, L-glutamine, and penicillin-streptomycin. Mycoplasma testing yielded negative results for passages 2–5, confirming the suitability of these cells for the experiment. The lentivirus plasmid pLV-CMV-CD19-T2A-Fluc-EF-1α-EGFP-T2A-Puro was constructed by initiating the transcription of CD19 cDNA fragments (nucleotides 66–1736; KJ890856.1)-T2A-Fluc and CopGFP-T2A-Puro under the control of the cytomegalovirus (CMV) and EF-1α promoters using overlapping PCR. The lentivirus was produced following an established protocol, and NALM6-GL cancer cells were selected for stable expression of GFP and luciferase. For the production of ASNS-overexpressing NALM6-GL cells, the lentivirus plasmid pLV-CMV-GFP-Fluc-ASNS-PGK-Puro-WPRE was used following a previously described protocol with ASNS cDNA (nucleotides 179–1864; NM27396.1). The expression of the ASNS protein in ASNS-OE NALM6-GL cancer cells was confirmed by immunoblot analysis. The oligos are included in Table S2.

### *In vitro* killing effect of CAR-T cells on the NALM6 cell line

The luciferase-based assay was optimized to assess the killing activity of CAR-T cells, as described in previous studies. NALM6-GL cells (target cells) were seeded in opaque white, flat-bottom 96-well plates (Coster, Shanghai, China) at a density of 50,000 cells in 100 μL of medium per well one day before the experiment. The cells were allowed to settle for 24 h at 37°C and 5% CO_2_. Modified CAR-T cells were then added to each well at the specified ratio and cultured for 24, 48, or 72 h in phenol-free RPMI-1640 medium supplemented with 10% FBS, 30 U/mL recombinant human IL-2, and 0.05 mM 2-mercaptoethanol. To measure the fluorescence intensity from luciferin, 10 μL of 150 μg/mL day-luciferin (Goldbio, LUCK-1G) was added to each well. The fluorescence intensity was measured within 10 min, and the background fluorescence was found to be negligible (<1% of the signal from wells containing only target cells). Each experiment was performed in triplicate. The percentage of cancer cell lysis was calculated using the following formula: (measured fluorescence intensity − minimum) / (maximum-minimum) × 100%, where the minimum was the fluorescence intensity of cells incubated with media alone, and the maximum was the fluorescence intensity of cells in 1% Triton X-100.

### L-ASPG treatment

L-ASPG was purchased from MedChemExpress (HY-P1923, Shanghai, China). L-ASPG was freshly dissolved and stored at 4°C until use in the coculture killing system with ASPG-KO CAR-T cells. The final concentration of L-ASPG for cotreatment was 0.5 IU/mL.^34^^,^^50^

### Flow cytometric analysis of CAR-T cells and cancer cells

Cells and mouse blood samples were collected, treated, and resuspended in magnetic activated cell sorting (MACS) buffer (PBS, 2% calf serum, and 1 mM EDTA). The specified cell surface markers were stained at 4°C for 30 min, followed by two washes with MACS buffer. To ensure comparable live cell counts across conditions, events were recorded from an equivalent fixed volume for all the samples. All gating strategies included exclusion of subcellular debris, singlet gating, and live/dead staining. All antibodies were used according to the manufacturer’s instructions. Cell analysis was performed using a CytoFLEX Flow Cytometer (Beckman Coulter, USA), and the data were analyzed using FlowJo software (FlowJo, USA). To identify CAR-T-cell subsets, cells were stained with anti-CD8-PE (BioLegend) at a 1:200 dilution and anti-DYKDDDDK (FLAG tag)-APC antibody (BioLegend) at a 1:200 dilution. NALM6-GL cancer cells were identified using the GFP-fluorescein isothiocyanate (FITC) channel. Each antibody was tested on cells that were known to be negative or positive for the target antigen. The memory cell phenotype of CAR-T cells was characterized by the expression of CD62L, CD45RA, CD44, CAR, and CD8. Exactly, we utilized an anti-CD45RA Percy/Cy5.5 antibody (BioLegend) at a 1:150 dilution and an anti-CD62L BV421 antibody (BioLegend) at a 1:200 dilution to explore the differentiation of Tscm (CD45RA^+^ CD62L^+^ CD8^+^ CAR^+^), Tcm (CD45RA^−^ CD62L^+^ CD8^+^ CAR^+^), Tem (CD45RA^−^ CD62L^−^ CD8^+^ CAR^+^), and Temra (CD45RA^+^ CD62L^−^ CD8^+^ CAR^+^), which were shown as percentages.

### Degranulation and intracellular cytokine production assay

The intracellular levels of granzyme B, IFN-γ, and TNF-α were measured following the coculture of effector CAR-T cells and target NALM6-GL cancer cells. On the day of the experiment, the cells were harvested and mixed in 200 μL of media containing the protein transport inhibitors brefeldin (10 μM) and monensin (2 μM). The culture supernatant was removed by centrifugation, and the cells were resuspended by live/dead near-infrared viability staining. After a 5-min incubation at room temperature, the cells were resuspended in 150 μL of fixation/permeabilization buffer (eBiosciences) for 30 min at room temperature. An anti-IFN-γ-PE/Cy7 antibody (BioLegend) at a 1:150 dilution, an anti-TNF-α-FITC (BioLegend) antibody at a 1:100 dilution, and an anti-granzyme B-PE antibody at a 1:200 dilution (BioLegend) were used to assess the function of CAR-T cells following coculture. CAR-T cells were identified by staining with an anti-CD8-PE antibody (BioLegend) at a 1:200 dilution and anti-DYKDDDDDK (FLAG tag)-APC antibody (BioLegend) at a 1:200 dilution. Distinct populations of cells expressing intracellular granzyme B, IFN-γ, and TNF-α were visualized using FlowJo software (FlowJo, USA).

### Apoptosis assay

Apoptosis was assessed by staining with Annexin V/propidium iodide following the coculture of effector CAR-T cells and targeting NALM6-GL cancer cells. On the day of the experiment, the cells were centrifuged and resuspended in 1× Annexin V binding buffer containing 5 μL of Annexin V-APC working solution and 10 μL of propidium iodide staining solution. The plate was subsequently incubated at 37°C for 15 min, after which 200 μL of 1× binding buffer was added to each well. Analysis of distinct populations of apoptotic cells was conducted following the exclusion of subcellular debris, singlet gating, and live/dead staining in CAR-T cells and NALM6-GL cancer cells.

### Proliferation assays

Cell proliferation was assessed by incubating CAR-T cells with 5 μM carboxyfluorescein succinimidyl ester (CFSE) staining solution following the manufacturer’s protocol (Thermo Fisher Scientific). After staining, the cells were washed three times and cultured in 96-well flat-bottomed plates for 5 days. Subsequently, the cells were collected in 200 μL of MACS buffer for the proliferation assay. After excluding subcellular debris, applying singlet gating, and conducting live-dead staining in CAR-T cells, distinct proliferation populations were visualized as mean fluorescence intensity.

### 2-NBDG glucose uptake assay

The 2-NBDG glucose uptake assay was done by incubating CAR-T cells with 2-NBDG reagent (Thermo Fisher Scientific) at a final concentration of 200 μg/mL at 37°C for 60 min, according to the manufacturer’s protocol. Afterward, the cells were collected in 200 μL of MACS buffer, and the populations of 2-NBDG-FITC+ cells were visualized as histograms after excluding subcellular debris, applying singlet gating, and conducting live/dead staining of the CAR-T cells.

### Immunoblot analysis

We analyzed NALM6 cancer cell lysates using standard SDS-PAGE. The lysates were probed with primary antibodies and horseradish peroxidase-conjugated secondary antibodies and visualized using the western enhanced chemiluminescence method. The following antibodies were used: anti-actin (GB15001, Servicebio, Shanghai, China) and anti-ASNS (ab40850; Abcam, USA).

### RNA isolation and RT-qPCR

Total RNA was isolated using TRIzol reagent (Life Technologies) and used as the template for preparing cDNA using HiScript II Reverse Transcriptase (R201, Vazyme, Shanghai, China) following the manufacturer’s instructions. The primers used for RT-qPCR are listed in Table S3. RT-qPCR was performed using the SYBR Green Mix Kit (A57155, SYBR Green, Invitrogen, USA) on a StepOnePlus Real-Time System (Invitrogen, USA), with human β-actin serving as the endogenous control.

### Xenogenic mouse models

The animal experimental designs and protocols were reviewed and approved by the Institutional Animal Care and Use Committee of Shanghai Sixth People’s Hospital, which is affiliated with the Shanghai Jiao Tong University School of Medicine (protocol no. 2021-0697). The experimental mice were housed in the specific pathogen-free area of the animal center, following a 12-h light-dark cycle, with ad libitum access to food and water as per specific instructions. NOD scid gamma(M-NSG, NOD.Cg-*Prkdc*^*scid*^*Il2rg*^*em1Smoc*^) mice aged 6–8 weeks were from Shanghai Model Organisms Center (Shanghai, China). After 1 week of acclimatization, the mice were administered 1 × 10^6^ NALM6-GL cells via tail vein injection. Starting on day 2, recombinant humanized IL-2 was administered via intraperitoneal injection at 1 μg per mouse every other day. Cancer-bearing mice received 2 × 10^6^ anti-CD19 CAR-T cells or modified CAR-T cells via tail vein injection on days 3, 7, and 10. Animals displaying signs of illness or distress were then euthanized. Survival analysis was performed by recording the euthanization time, and each group contained at least 10 mice. For the analysis of immunophenotyping with flow cytometry, another experiment was done by collecting the blood samples via retro-orbital bleeding on day 21 or day 28. The resident cancer cells and CAR-T cells were enriched after cleaning the red blood cells using ACK lysing buffer (Thermo Fisher Scientific).

### Statistical analysis

The statistical tests and details of the experiments are provided in the figure panels, legends, and supplemental information. The experiments were independently performed at least in triplicate. Data analyses were conducted, and the data were exported to GraphPad Prism for visualization. Quantitative data are presented as the mean ± standard deviation (SD). For experiments involving two or more independent groups, the data were analyzed using two-sample t tests and one-way or two-way analysis of variance (ANOVA), respectively. *p* values were adjusted for multiple comparisons using Sidak’s multiple comparison test. In experiments with the same samples treated under various conditions, such as different E:T ratios, mixed-effect modeling was utilized to test differences among the E:T ratios. The data normality assumption was assessed using residual plots, and outliers identified on the residual plots were excluded from the data analysis. Mouse survival data were analyzed using the Kaplan-Meier method, and survival probabilities among groups were compared using a log rank test. *p* values are denoted with asterisks as follows: ∗*p* < 0.05; ∗∗*p* < 0.01; ∗∗∗*p* < 0.001; and ∗∗∗∗*p* < 0.0001. These data are presented in Tables S4–S36.

## Data availability

The data used in this study are available upon request from the lead contact.

## Acknowledgments

This study involved human samples that were anonymously coded following local ethical guidelines, as stipulated by the Declaration of Helsinki. Written informed consent was obtained from all healthy donors with approval from the Ethics Committee of Shanghai Sixth People’s Hospital Affiliated Shanghai Jiao Tong University School of Medicine (no. 2021-YS-0215), and written informed consent was obtained before participation. The animal experimental designs and protocols were reviewed and approved by the Institutional Animal Care and Use Committee at Shanghai Sixth People’s Hospital Affiliated Shanghai Jiao Tong University School of Medicine (Protocol No.: 2021-0697). This work was supported by grants from the 10.13039/501100001809National Natural Science Foundation of China (no. 82272925, 81872494, and 82274151), Shanghai Pujiang Talent Project (no. 21PJ1411900), and 10.13039/501100008233Shanghai Jiao Tong University School of Medicine Research Physician Program (no. 20240815). We thank Shanghai Jiao Tong University School of Medicine for their help with the flow cytometry experiment and Instrumental Analysis Center Shanghai Jiao Tong University for their help with the experimental analysis.

## Author contributions

Conceptualization: Q.Y. and C.G.; data curation: Z.X. and Q.Y.; funding acquisition: C.G. and Q.Y.; methodology: X.Z., L.H., D.B., J.H., L.Y., S.L., R.G., B.X., Y.T., and Z.X.; project administration: Q.Y. and C.G.; resources: Y.Z., J.Z., and C.G.; supervision: C.G. and Y.H.; validation: Q.Y., L.H., and X.Z.; visualization: Q.Y., D.B., L.H., and X.Z.; writing – original draft preparation: Q.Y., Z.X., X.Z., L.H., J.H., D.B., and C.G.

## Declaration of interests

The authors declare no competing interests.

## Supplemental information

Document S1. Figures S1–S4 and Tables S1–S36

Document S2. Article plus supplemental information
