# Supplementary material for: Asparaginase enhances CAR-T cell antitumor immunity by asparagine metabolic reprogramming and central memory induction in ALL
Source: Mol Ther. 2025 Aug 12;33(11):5572–90. doi: 10.1016/j.ymthe.2025.08.019 (PMC12628164; doi:10.1016/j.ymthe.2025.08.019)
Supplement: Document S1. Figures S1–S4 and Tables S1–S36 [file mmc1.pdf]

YMTHE, Volume *33*

Supplemental Information

Asparaginase enhances CAR-T cell antitumor immunity by asparagine metabolic reprogramming and central memory induction in ALL

Xinting Zhu, Leng Han, Dingyuan Bai, Lei Yi, Yonghong Zhao, Shuaibing Liu, Run Gan, Bo Xin, Yixing Tu, Jianping Zhang, Yonglong Han, Juan Hao, Zixue Xuan, Cheng Guo, and Quanjun Yang

Supplemental Tables

Table S1: Oligos for the cloning of ASPG into the CAR-T vector.

Gene	NCBI Reference Sequence	F1-ASPG	R1-ASPG
ASPG	NM_001080464.3	tgctaacatgcggtgacgtcgaggagaatcctggcccaGAATTCatggcgcgcgcggtgg	gtaatccagaggttgattgtcgacttaacgcgtttagacaccaggcagcacttctgggcatgggg

Table S2, Oligos for the cloning of ASNS into the pLVX-EnCMV-AcGFP1-Linker-Fluc-PGK-Puro-WPRE vector.

Gene	NCBI Reference Sequence	F1-ASNS	R1-ASNS
ASNS	NM_133436.3	gggcagaggaagtctgctaacatgcggtgacgtcgaggagaatcctggcccaGAATTCatgtgtggcatttgggcgctgtttggca	CCTACCCGGTAGAATTATGATCAGTTATCTAGATCCGGTctaagctttgacagctgacttgtagtgggtcagcgt
ASNS	NM_133436.3	GGCCAAGAAGGGCGGAAAGATCGCCGTGGATCCggcagtggagagggcagaggaagtctgctaacatgcggtgacgtcgaggagaatcctggcccaGAATTCatgtgtggcatttgggcgctgtttggca	GAATTATGATCAGTTATCTAGATCCGGTctaagctttgacagctgacttgtagtgggtcagcgt

Table S3: Primers for real-time qRT–PCR.

Gene	NCBI Reference Sequence	Forward primer	Reverse primer	Product size (bp)
ASNS	NM_133436.3	gcgaccaaaagaagccttca	gcatccagtaatggctcagc	224
ASPG	NM_001080464.3	aggattctctacaccgtgct	gcatgaaggacagcatcgag	178
INFG	NM_000619.3	ctgttactgccaggacccat	ctcttttggatgctctggtcat	216
IL6	NM_000600.5	cctgatccagttcctgcaga	gaactccttaaagctgcgca	157
IL2	NM_000586.4	acaaacagtgcacctacttca	tcagttctgtggccttcttg	169
GZMB	NM_004131.6	cgacccagcagtttatccct	gtgtgagtgttttcccaggg	224
PRF1	NM_005041.6	acggatgcctatgtgaagct	agacttgggagcctgatcac	207
ACTB	NM_001101.5	tggcaccacaccttctacaa	ccagaggcgtacagggatag	182

Table S4: Raw statistics data of Fig 1D. Cancer cell lysis assay of effector CAR-T cells to target cancer cells (E:T) at ratios of 1:1, 0.5:1, 0.25:1, and 0.1:1 after co-culture for 24 hours (D) and 72 hours (E). Two-way ANOVA was used for statistical analysis, and Sidak's multiple comparisons test was used for comparisons between two groups.

	NALM6-GL					ASNS-OE NALM6-GL				
E:T=1:1	74.85	71.67	72.2	75.42	78.25	51.51	70.42	55.51	66.02	61.93
E:T=1:2	61.84	56.65	54.89	61.07	56.36	48.65	42.56	53.71	47.52	43.03
E:T=1:8	40.67	39.69	34.96	40.89	31.56	30.24	32.76	24.28	29.62	28.76
E:T=1:10	18.38	19.92	7.23	14.6	14.38	10.11	6.23	12.12	16.51	10.88

Table S5: Raw statistics data of Fig 1E. Cancer cell lysis assay of effector CAR-T cells to target cancer cells (E:T) at ratios of 1:1, 0.5:1, 0.25:1, and 0.1:1 after co-culture for 24 hours (D) and 72 hours (E). Two-way ANOVA was used for statistical analysis, and Sidak's multiple comparisons test was used for comparisons between two groups.

	NALM6-GL					ASNS-OE NALM6-GL				
E:T=1:1	77.98	75.1	83.54	78.81	79.45	62.72	60.47	78.13	58.21	59.71
E:T=1:2	62.72	60.47	68.13	68.21	60.37	51.41	42.65	43.45	39.62	45.67
E:T=1:8	48.89	43.38	36.62	45.52	34.87	28.80	25.67	29.24	14.84	26.28
E:T=1:10	20.93	13.52	34.95	15.10	24.69	11.21	16.56	10.14	7.46	6.68

Table S6: Raw statistics data of Fig 1G. Grouped histogram (G) showing the resident CAR-T cells and cancer cells at an E:T ratio of 1:1 after co-culture for 72 hours. Two-way ANOVA was used for statistical analysis, and Sidak's multiple comparisons test was used for comparisons between two groups. The number of samples with biological replicates is shown as dots in a bar graph.

	Coculture of NALM6-GL					Coculture of ASNS-OE NALM6-GL				
E:T=1:1	82.6	81.6	83.6	84.2	82.1	63.8	64.2	60.2	61.5	62.7
E:T=1:2	68.8	61.4	64.5	65.7	68.5	50.8	49.1	51.6	48.7	44.7
E:T=1:8	57.7	54.9	59.6	52.7	54.3	45	42.5	46.7	47.2	43.9
E:T=1:10	30.9	32.4	34.5	36.1	32.5	11.7	9.78	11.9	10.5	12.2

	Coculture of NALM6-GL					Coculture of ASNS-OE NALM6-GL				
E:T=1:1	15.3	14.6	14.2	14.9	15.8	34.6	35.7	36.2	37.1	36.4
E:T=1:2	30.7	31.2	29.4	28.7	31.2	46.9	45.8	47.1	47.8	46.5
E:T=1:8	41.2	42.5	44.3	41.5	42.1	52.9	53.2	54.7	51.4	55.7
E:T=1:10	68.3	67.4	69.1	66.4	67.5	87.3	81.4	80.2	79.4	79.6

Table S7: Raw statistics data of Fig 1I. Grouped histogram (I) showing the percentage of intracellular Granzyme B-positive CAR-T cells at an E:T ratio of 1:1 after co-culture for 72 hours. Two-way ANOVA was used for statistical analysis, and Sidak's multiple comparisons test was used for comparisons between two groups. The number of samples with biological replicates is shown as dots in a bar graph.

	CoCulture of NALM6-GL					Coculture of ASNS-OE NALM6-GL				
24 Hours	23.4	24.5	21.6	27.4	22.5	19.9	19.7	21.3	20.7	18.6
48 Hours	39.7	37.8	35.9	37.4	38.6	18.4	18.9	19.5	21.6	22.7

Table S8: Raw statistics data of Fig 1K. Grouped histogram (K) showing the percentage of intracellular IFN-γ and TNF-α-positive CAR-T cells at an E:T ratio of 1:1 after co-culture for 72 hours. Two -way ANOVA was used for statistical analysis, and Sidak's multiple comparisons test was used for comparisons between two groups. The number of samples with biological replicates is shown as dots in a bar graph.

	CoCulture of NALM6-GL					Coculture of ASNS-OE NALM6-GL				
24 Hours	22.6	22.9	21.4	20.7	19.6	19.4	16.7	17.8	17.1	18.9
48 Hours	25.6	26.7	27.4	25.1	27.3	16.1	16.4	15.9	17.4	16.6

	CoCulture of NALM6-GL					Coculture of ASNS-OE NALM6-GL				
24 Hours	15.2	15.4	14.8	15.9	14.1	3.13	3.24	3.37	3.16	3.39
48 Hours	15.9	16.2	16.3	14.8	16.9	2.65	2.48	2.67	2.34	2.5

Table S9: Raw statistics data of Fig 2B. Histogram showed CFSE-based cell proliferation assay revealing the distinct expansion capacity of ASPG-OE CAR-T cells. A two-sample t-test was used for statistics. The number of samples with biological replicates is shown as dots in a bar graph.

ASPG-OE CAR-T	CAR-T
87.7	74.7
86.4	72.9
91.2	76.3

Table S10: Raw statistics data of Fig 2C. Histogram showed a 2-NBDG based glucose uptake assay revealing the distinct metabolic adaptation of ASPG-OE CAR-T cells. A two-sample t-test was used for statistics. The number of samples with biological replicates is shown as dots in a bar graph.

ASPG-OE CAR-T	CAR-T
72.3	65.8
69.3	64.1
76.2	66.9

Table S11: Raw statistics data of Fig 2D. The cancer cell lysis assay of effector ASPG-OE CAR-T cells to target ASNS-OE NALM6-GL cancer cells (E:T) at ratios of 1:1, 0.5:1, 0.25:1, and 0.1:1 after co-culture for 24 hours (up) and 72 hours (down). Two-way ANOVA was used for statistical analysis, and Sidak's multiple comparisons test was used for comparisons between two groups.

	ASPG-OE CAR-T					CAR-T					Mock CAR-T				
E:T=1:1	79.69	76.03	83.59	78.94	79.56	69.02	67.05	71.05	67.93	67.78	14	14.3	15	11.8	14.6
E:T=1:2	56.9	63.23	63.64	62.85	57.82	53.08	44.42	52.35	46.06	50.41	10.5	12.6	14.1	12.3	12.3
E:T=1:8	47.17	41.36	36.9	44.1	39.17	20.25	33.62	24.87	23.47	15.4	8.83	9.21	10.28	10.8	9.44
E:T=1:10	28.6	20.28	26.75	30.85	31.34	10.43	23.96	14.83	16.86	17.19	6.33	4.17	3.99	4.4	5.34

	ASPG-OE CAR-T					CAR-T					Mock CAR-T				
E:T=1:1	91.98	88.54	83.95	85.81	87.13	76.5	84.02	74.43	71.46	73.62	14.1	16.3	15.2	11.8	14.9
E:T=1:2	82.49	73.81	72.67	72.97	78.89	60.07	57.06	55.32	57.58	64.84	10.6	12.8	14.9	11.5	12.4
E:T=1:8	58.86	60.36	51.42	51.86	50.27	44.55	46.21	30.55	21.15	40.89	8.97	9.41	10.45	11	9.62
E:T=1:10	33.12	35.61	22.93	28.61	35.3	25.2	14.85	21.38	20.65	17.64	3.57	4.25	3.17	4.62	3.87

Table S12: Raw statistics data of Fig 2F. Grouped histogram (F) showed the intracellular Granzyme B-positive ASPG-OE CAR-T cells and control CAR-T cells percentage at an E:T ratio of 1:1 after co-culture for 72 hours. Two-way ANOVA was used for statistical analysis, and Sidak's multiple comparisons test was used for comparisons between two groups. The number of samples with biological replicates is shown as dots in a bar graph.

	CAR-T					ASPG-OE CAR-T				
24 Hours	20.5	20.9	19.4	18.6	21.7	22.7	21.4	22.9	21.6	20.4
72 Hours	21.3	21.6	20.6	19.8	17.9	29.3	30.4	28.6	27.9	31.7

Table S13: Raw statistics data of Fig 2H. Grouped histogram (H) showed the intracellular IFN-γ and TNF-α-positive ASPG-OE CAR-T cells and control CAR-T cells percentage at an E:T ratio of 1:1 after co-culture for 72 hours. Two-way ANOVA was used for statistical analysis, and Sidak's multiple comparisons test was used for comparisons between two groups. The number of samples with biological replicates is shown as dots in a bar graph.

	CAR-T					ASPG-OE CAR-T				
24 Hours	10.2	9.87	11.6	12.4	10.7	19.8	17.6	18.6	20.4	19.4
72 Hours	12.6	13	9.65	13.7	14.8	22.6	24.6	23.7	21.6	22.4

	CAR-T					ASPG-OE CAR-T				
24 Hours	2.88	2.68	2.94	2.67	2.54	13.9	14.8	12.4	9.78	11.6
72 Hours	2.82	2.45	2.49	2.71	2.89	14.5	14.7	15.2	11.7	13.6

Table S14: Raw statistics data of Fig 2J. Grouped histogram (J) showed the memory phenotype in the CAR+ CD8+ T cells of ASPG-OE CAR-T cells and control CAR-T cells at an E:T ratio of 1:1 after co-culture for 72 hours. The Tscm (CD45RA+ CD62L+), Tcm (CD45RA- CD62L+), Tem (CD45RA- CD62L-), and Temra (CD45RA+ CD62L-) were shown as percentages. Two-way ANOVA was used for statistical analysis, and Sidak's multiple comparisons test was used for comparisons between two groups. The number of samples with biological replicates is shown as dots in a bar graph.

	CAR-T					ASPG-OE CAR-T				
Tscm	0.0098	0.0045	0.0034	0.0059	0.0671	0.37	0.48	0.29	0.48	1.12
Tcm	4.42	4.18	4.85	5.24	3.18	32.9	31.91	30.82	30.69	30.3
Tem	78.4	72.6	79.4	70.4	73.5	31.3	30.5	37.2	33.4	32.7
Temra	17.1	18.2	19.7	16.4	15.3	35.5	34.7	33.6	36.1	34.2

Table S15: Raw statistics data of Fig 3C. The mRNA expression of ASPG in the three groups of ASPG-KO CAR-T cells was analyzed by relative quantitative PCR. One-way ANOVA was used for statistical analysis, and Sidak's multiple comparisons test was used for comparison between two groups. The number of samples with biological replicates is shown as dots in a bar graph.

NTC	sg_1	sg_2	sg_3
1.05	0.76	0.54	0.67
1.08	0.69	0.57	0.62
0.87	0.72	0.58	0.58

Table S16: Raw statistics data of Fig 3D. Histogram showed CFSE based cell proliferation assay revealing distinct expansion capacity of the three groups of ASPG-KO CAR-T cells. One-way ANOVA was used for statistical analysis, and Sidak's multiple comparisons test was used for comparison between two groups. The number of samples with biological replicates is shown as dots in a bar graph.

NTC	sg_1	sg_2	sg_3
77.1	40.1	22.9	26.9
72.3	42.6	23.4	25.8
74.8	41.7	20.7	29.7

Table S17: Raw statistics data of Fig 3E. Histogram showed 2-NBDG based glucose uptake assay revealing distinct metabolic adaptation of the three groups of ASPG-KO CAR-T cells. One-way ANOVA was used for statistical analysis, and Sidak's multiple comparisons test was used for comparison between two groups. The number of samples with biological replicates is shown as dots in a bar graph.

NTC	sg_1	sg_2	sg_3
66.1	61.5	41.2	51.1
60.4	62.4	40.7	53.7
63.7	58.9	42.4	55.6

Table S18: Raw statistics data of Fig 3F. The cancer cell lysis assay of effector sg-2 of ASPG-KO CAR-T cells to target ASNS-OE NALM6-GL cancer cells (E:T) at a ratio of 1:1, 0.5:1, 0.25:1, and 0.1:1 from co-culture for 24 hours (up) and 72 hours (down). Two-way ANOVA was used for statistical analysis, and Sidak's multiple comparisons test was used for comparison between two groups.

	ASPG-KO CAR-T					CAR-T				
E:T=1:1	45.91	49.41	45.36	48.41	46.3	61.27	62.8	64.46	61.78	70.69
E:T=1:2	23.74	37.77	31.71	34.6	33.87	45.02	49.52	41.24	35.74	50.31
E:T=1:8	21.88	16.6	33.96	19.22	22	37.58	25.53	21.55	29.93	34.04
E:T=1:10	4.26	17.29	10.62	7.18	3.19	19.85	9.97	11.32	17.48	20.84

	ASPG-KO CAR-T					CAR-T				
E:T=1:1	54.03	45.11	50.8	49.29	47.73	75.53	73.91	72.28	76.06	77.83
E:T=1:2	37.23	34.28	29.93	33.75	35.49	68.16	69.93	55.98	72.07	62.04
E:T=1:8	15.58	26.4	19.45	18.15	12.02	65.7	52.55	53	55	47.38
E:T=1:10	15.65	13.39	17.13	5.96	16.55	43.46	41.18	40.34	34.11	49.53

Table S19: Raw statistics data of Fig 3H. Histogram showed the intracellular Granzyme B positive percentages from the three groups of ASPG-KO CAR-T cells at E:1 ratio of 1:1 after co-culture for 72 hours. One-way ANOVA was used for statistical analysis, and Sidak's multiple comparisons test was used for comparison between two groups. The number of samples with biological replicates is shown as dots in a bar graph.

NTC	sg_1	sg_2	sg_3
20.9	7.13	4.53	6.74
21.5	7.92	4.26	6.78
20.1	6.48	4.17	7.24

Table S20: Raw statistics data of Fig 3J. Histogram showed the intracellular IFN-γ and TNF-α positive percentages from the three groups of ASPG-KO CAR-T cells at E:1 ratio of 1:1 after co-culture for 72 hours. One-way ANOVA was used for statistical analysis, and Sidak's multiple comparisons test was used for comparison between two groups. The number of samples with biological replicates is shown as dots in a bar graph.

NTC	sg_1	sg_2	sg_3
17.8	10.2	7.64	10.1
17.4	10.7	7.19	9.49
18.3	9.64	8.21	9.38

NTC	sg_1	sg_2	sg_3
15.6	3.35	3.61	3.07
14.8	3.37	3.49	3
15.7	3.27	3.37	2.89

Table S21: Raw statistics data of Fig 3L, Grouped histogram showed the memory phenotype in the CAR+ CD8+ T cells percentages from the three groups of ASPG-KO CAR-T cells at E:1 ratio of 1:1 after co-culture for 72 hours. The Tcm (CD45RA- CD62L+), Tem (CD45RA- CD62L-), and Temra (CD45RA+ CD62L-) were shown as percentages. Two-way ANOVA was used for statistical analysis, and Sidak's multiple comparisons test was used for comparison between two groups. The number of samples with biological replicates is shown as dots in a bar graph.

	**NTC**			**sg_1**			**sg_2**			**sg_3**		
Tcm	21.1	21.6	21.9	19.7	18.6	20.4	3.18	3.28	4.12	19	18.4	20.8
Tem	76.0	74.9	75.8	79	78.6	68.9	96.8	94.6	95.2	79.7	75.6	77.9
Temra	2.48	3.07	1.50	0.84	2.32	10.25	0.94	1.90	0.49	1.09	5.82	0.82

Table S22: Raw statistics data of Fig 4B. Overall survival of mice bearing ASNS-OE NALM6-GL cancer after receiving CAR-T and ASPG-OE CAR-T cell therapy. Survival data were analyzed using the Kaplan-Meier method, and survival probabilities among groups were compared using a log-rank test.

DPI	Mock CAR-T	CAR-T	ASPG-OE CAR-T
16	1		
18	1		
20		1	
21	1		
21	1		
22	1		
23	1		
25	1		
25		1	
26	1		
27		1	
28		1	
29	1		
30		1	
31		1	
32	1		
34		1	
34		1	
35			1
39		1	
44			1
45			1
46		1	

47	1
48	1
48	1
50	1
51	1
56	0
56	0

Table S23: Raw statistics data of Fig 4D. Histogram showing resident ASNS-OE NALM6-GL cancer cells and CAR-T cells in the blood of mice at day 28 after receiving CAR-T and ASPG-OE CAR-T therapy. One-way ANOVA was used for statistical analysis, and Sidak's multiple comparisons test was used for comparison between two groups. The number of samples with biological replicates is shown as dots in a bar graph.

Mock CAR-T	CAR-T	ASPG-OE CAR-T
10.3	7.74	1.28
10.1	7.15	1.21
9.87	8.24	1.35
9.64	8.16	1.42
10.2	7.94	1.18

Mock CAR-T	CAR-T	ASPG-OE CAR-T
0.022	1.47	12
0.012	1.26	11.2
0.061	1.46	10.6
0.02	1.54	14.2
0.01	1.66	13.7

Table S24: Raw statistics data of Fig 4F. Grouped histogram showing the memory phenotype of CAR-T cells in the blood of mice at day 28 after receiving CAR-T and ASPG-OE CAR-T therapy. Tcm (CD45RA- CD62L+), Tem (CD45RA- CD62L-), and Temra (CD45RA+ CD62L-) are shown as percentages. Two-way ANOVA was used for statistical analysis, and Sidak's multiple comparisons test was used for comparison between two groups. The number of samples with biological replicates is shown as dots in a bar graph.

	ASPG-OE CAR-T					CAR-T				
Tcm	34.2	32.4	33.1	34.2	35.1	5.28	6.41	5.19	6.92	7.23
Tem	53.4	51.3	52.4	53	52.8	68.9	56.5	67.2	60.2	63.2
Temra	12.4	11.9	12.4	11.6	10.9	25.8	36.8	27.12	31.96	29.51

Table S25: Raw statistics data of Fig 4G. Overall survival of mice bearing ASNS-OE NALM6-GL cancer after receiving CAR-T and SPG-KO CAR-T cell therapy. Survival data were analyzed using the Kaplan-Meier method, and survival probabilities among groups were compared using a log-rank test.

DPI	CAR-T	ASPG-KO CAR-T
14		1
16		1
19		1
20	1	
22		1
23	1	
25		1
25	1	
26		1
27		1
28	1	
29		1
31	1	
32		1
33		1
33		1
34	1	
34	1	
35	1	
39	1	
42	1	
45	1	

Table S26: Raw statistics data of Fig 4I. Histogram (I) showing resident ASNS-OE NALM6-GL cancer cells and CAR-T cells in the blood of mice at day 28 after receiving CAR-T and ASPG-KO CAR-T therapy. One-way ANOVA was used for statistical analysis, and Sidak's multiple comparisons test was used for comparison between two groups. The number of samples with biological replicates is shown as dots in a bar graph.

CAR-T	ASPG-KO CAR-T
6.41	10.5
6.27	10.3
5.97	9.68
6.79	10.6
8.12	9.47

CAR-T	ASPG-KO CAR-T
1.59	0.49
1.06	0.42
1.27	0.37
1.39	0.59
1.74	0.51

Table S27: Raw statistics data of Fig 5A. Grouped histogram showing the cancer cell lysis assay of effector ASPG-modified CAR-T cells against ASNS-OE NALM6-GL cancer cells (E:T) at ratios of 1:1, 0.5:1, 0.25:1, and 0.1:1 after co-culture for 24 hours. Two-way ANOVA was used for statistical analysis, and Sidak's multiple comparisons test was used for comparisons between two groups. The number of samples with biological replicates is shown as dots in a bar graph.

	ASPG-KO CAR-T					CAR-T					ASPG-OE CAR-T				
E:T=1:1	60.71	55.45	50.36	63.86	56.95	77.27	78.77	85.46	77.63	71.66	88.59	89.19	96.98	93.97	90.32
E:T=1:2	42.93	48.43	45.19	41.81	33.67	64.02	59.88	61.33	58.95	62.25	70.38	73.01	78.98	68.75	65.54
E:T=1:8	30.28	30.28	20.29	19.21	26.45	31.99	46.34	41.70	46.05	36.54	48.35	47.56	55.64	45.5	51.24
E:T=1:10	19.87	0.66	10.52	10.76	9.17	33.13	22.17	25.01	32.25	27.41	30.84	36.42	34.04	28.02	37.16

Table S28: Raw statistics data of Fig 5C. Grouped histogram showing the percentage of residual ASNS-OE NALM6-GL cells at an E:T ratio of 1:1 after co-culture for 24 hours. Two-way ANOVA was used for statistical analysis, and Sidak's multiple comparisons test was used for comparisons between two groups. The number of samples with biological replicates is shown as dots in a bar graph.

	ASPG-KO CAR-T					CAR-T					ASPG-OE CAR-T				
E:T=1:1	9.99	12.5	16.4	7.89	14.6	5.62	5.19	5.47	6.24	5.93	2.53	2.64	3.67	3.02	3.42
E:T=1:2	29.7	31.2	26.7	35.4	32.4	14.8	15.6	17.2	13.7	13.9	8.48	9.56	7.48	7.28	9.51
E:T=1:8	45.2	51.2	47.8	46.2	51.3	32.7	39.4	32.7	31.4	28.2	15.1	16.2	15.4	14.7	15.8
E:T=1:10	79.6	81.2	75.6	82.6	77.4	51.8	59.4	53.6	49.7	53.7	28.3	32.5	30.6	29.7	31.4

Table S29: Raw statistics data of Fig 5D. Grouped histogram showing the percentage of CAR-T cells responsible for killing (D) at an E:T ratio of 1:1 after co-culture for 24 hours. Two-way ANOVA was used for statistical analysis, and Sidak's multiple comparisons test was used for comparisons between two groups. The number of samples with biological replicates is shown as dots in a bar graph.

	ASPG-KO CAR-T					CAR-T					ASPG-OE CAR-T				
E:T=1:1	87.7	82.5	80.6	84.3	86.1	88.8	87.6	89.4	81.7	90.7	95.9	94.3	91.6	90.3	92.8
E:T=1:2	67.5	62.3	62.4	67.1	61.4	82.8	81.6	86.4	89.1	88.9	89.3	84.6	85.7	85.7	89.4
E:T=1:8	49.2	50.3	48.2	46.1	50.3	62.4	59.7	57.9	66.7	64.3	80.1	81.9	77.9	82.6	81.6
E:T=1:10	18.9	18.9	20.6	21.2	23.7	45.6	50.7	42.8	46.7	44.9	70.2	69.4	71.6	74.6	68.9

Table S30: Raw statistics data of Fig 6A. Grouped histogram showed the apoptosis percentages of NALM6-GL cancer cells from the ASPG modified BCAR-T cells treatment at E:1 ratio of 1:1 after co-culture for 24 hours. Two-way ANOVA was used for statistical analysis, and Sidak's multiple comparisons test was used for comparison between two groups. The number of samples with biological replicates is shown as dots in a bar graph.

	ASPG-KO CAR-T					CAR-T					ASPG-OE CAR-T				
Early apoptosis	13.9	14.6	15.4	13.4	12.7	12.4	12.8	11.3	9.87	10.2	17	16.7	19.2	20.3	14.5
Late apoptosis	42.6	49.2	41.4	40.6	39.7	26.7	27.2	29.6	30.3	27.8	60.6	57.8	62.3	59.6	60.4

Table S31: Raw statistics data of Fig 6B. Histogram showed the intracellular Granzyme B positive percentages. One-way ANOVA was used for statistical analysis, and Sidak's multiple comparisons test was used for comparison between two groups. The number of samples with biological replicates is shown as dots in a bar graph.

ASPG-KO CAR-T	CAR-T	ASPG-OE CAR-T
19.5	10.9	37.7
18.6	10.1	36.4
20.4	12.3	35.8

Table S32: Raw statistics data of Fig 6C. Histogram showed the intracellular IFN-γ and TNF-α positive percentages from the ASPG modified CAR-T cells. One-way ANOVA was used for statistical analysis, and Sidak's multiple comparisons test was used for comparison between two groups.

ASPG-KO CAR-T	CAR-T	ASPG-OE CAR-T
21.8	14.7	24.7
21.4	15.2	26.2
19.6	13.3	24.9

ASPG-KO CAR-T	CAR-T	ASPG-OE CAR-T
14.7	2.73	15.19
13.2	4.68	17.4
14.9	3.72	16.1

Table S33: Raw statistics data of Fig 6D. Grouped histogram showed the memory phenotype in the CAR+ CD8+ T cells from the ASPG modified CAR-T cells after culture for 14 days. The Tcm (CD45RA- CD62L+), Tem (CD45RA- CD62L-), and Temra (CD45RA+ CD62L-) were shown as percentages. Two-way ANOVA was used for statistical analysis, and Sidak's multiple comparisons test was used for comparisons between two groups.

	ASPG-KO CAR-T					CAR-T					ASPG-OE CAR-T				
Tcm	6.33	6.37	5.97	5.84	6.2	19.1	19.7	18.6	17.3	18.6	29.9	26.8	33.6	30.3	27.6
Tem	76.7	71.6	78.9	74.3	72.4	60.1	59.7	60.4	60.7	60.8	59.7	57.6	52.3	54.7	58.2
Temra	16.8	17.96	13.96	16.48	17.75	20.8	19.43	19.73	21.34	20.05	9.81	11.66	10.57	9.71	10.19

Table S34: Raw statistics data of Fig 7B. Overall survival of mice bearing NALM6-GL cancer after receiving CAR-T and ASPG-modified CAR-T cell therapy. Survival data were analyzed using the Kaplan-Meier method, and survival probabilities among groups were compared using a log-rank test.

DPI	Mock T	CAR-T	ASPG-KO CAR-T	ASPG-OE CAR-T
15	1			
17	1			
19	1		1	
20	1		1	
20	1			
21			1	
22	1			
23		1		
24	1		1	
24	1		1	
27		1		
28	1		1	
28	1			
29		1		
29		1		
30				1
31			1	
33		1		
34			1	
35			1	
36		1		
37				1

38			1
39	1		
40		1	
42	1		
43	1		
44			1
47			1
48	1		
52			1
56			1
57			1
61			1
63			1

Table S35: Raw statistics data of Fig 7D. Histogram showing resident NALM6-GL cancer cells and CAR-T cells in the blood of mice at day 21 after receiving CAR-T and ASPG modified CAR-T therapy.

Mock CAR-T	ASPG-KO CAR-T	CAR-T	ASPG-OE CAR-T
14.9	13.1	7.92	8.46
13.8	12.2	8.24	7.92
14.9	12.7	8.16	8.97
15.7	13.9	6.79	9.15
13.4	14.1	8.5	9.46

Mock CAR-T	ASPG-KO CAR-T	CAR-T	ASPG-OE CAR-T
0.041	0.26	1.18	6.81
0	0.36	1.26	6.47
0.035	0.37	1.59	7.29
0.021	0.42	1.47	6.98
0.215	0.29	1.33	6.79

Table S36: Raw statistics data of Fig 7F. Grouped histogram showing the memory phenotype of CAR-T cells in the blood of mice at day 21 after receiving CAR-T and ASPG-modified CAR-T therapy. Tcm (CD45RA- CD62L+), Tem (CD45RA- CD62L-), and Temra (CD45RA+ CD62L-) are shown as percentages.

	ASPG-KO CAR-T					CAR-T					ASPG-OE CAR-T				
Tcm	5.08	5.36	5.29	6.07	6.34	6.32	6.48	6.51	6.42	6.36	22.8	23.2	21.9	22.4	21.7
Tem	73.4	72.8	71.6	74.2	70.2	76.7	75.3	71.4	76.2	77.1	64.3	62.7	63.9	61.6	64.8
Temra	21.5	21.4	22.7	20.2	23.1	17	16.9	21.3	16.8	16.4	12.7	13.2	14.1	12.7	13.4

Supplemental figures

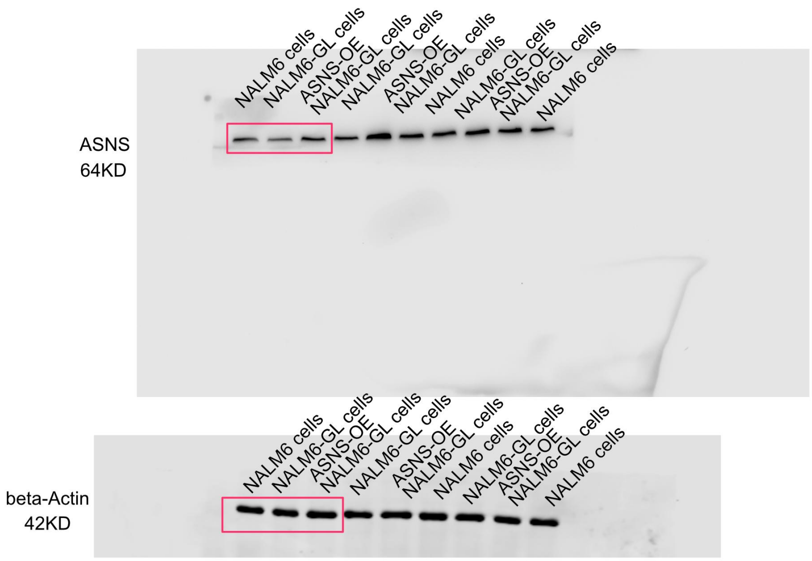

Figure S1: The orginal protein expression of ASNS was confirmed by immunoblotting.

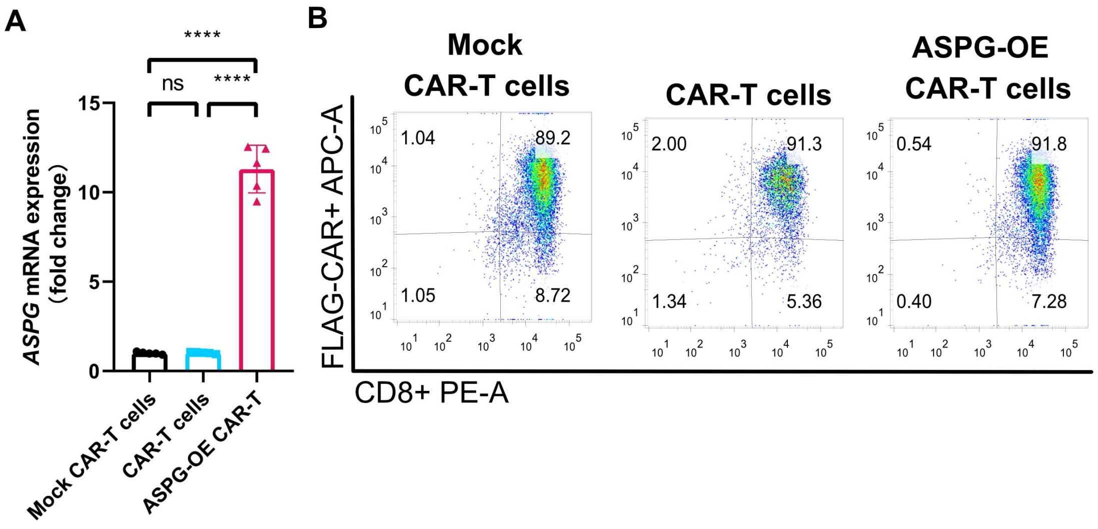

Figure S2:ASPG mRNA and robust CAR expression in the CAR-T cells. (A) The ASPG mRNA expression was determined by real time qPCR and the quantitative analysis was performed by comparing beta-action gene expression. One-way ANOVA was used for statistical analysis, and Sidak's multiple comparisons test was used for comparison between two groups. The number of samples with biological replicates is shown as dots in a bar graph. P-values are denoted with asterisks as follows: not significant (ns); *, p-value < 0.05; **, p-value < 0.01; ***, p-value < 0.001; ****, p-value < 0.0001. (B) The CAR expression was reflected by Flag-tag that was determined by flow cytometry of expression.

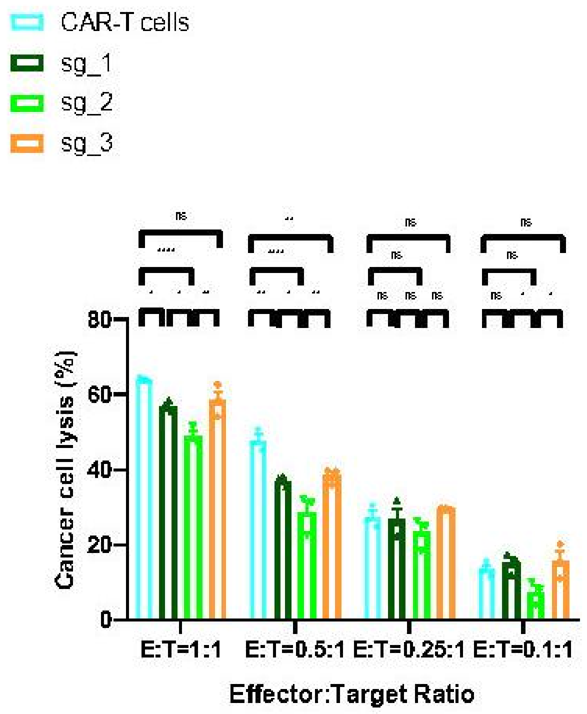

Figure S3：The cancer cell lysis assay of three sgRNA of ASPG-KO CAR-T cells to target ASNS-OE NALM6-GL cancer cells (E : T) at a ratio of 1:1, 0.5:1, 0.25:1, and 0.1:1 from co-culture for 24 hours. Two-way ANOVA was used for statistical analysis, and Sidak's multiple comparisons test was used for comparison between two groups. P-values are denoted with asterisks as follows: not significant (ns); *, p-value < 0.05; **, p-value < 0.01; ***, p-value < 0.001; ****, p-value < 0.0001.

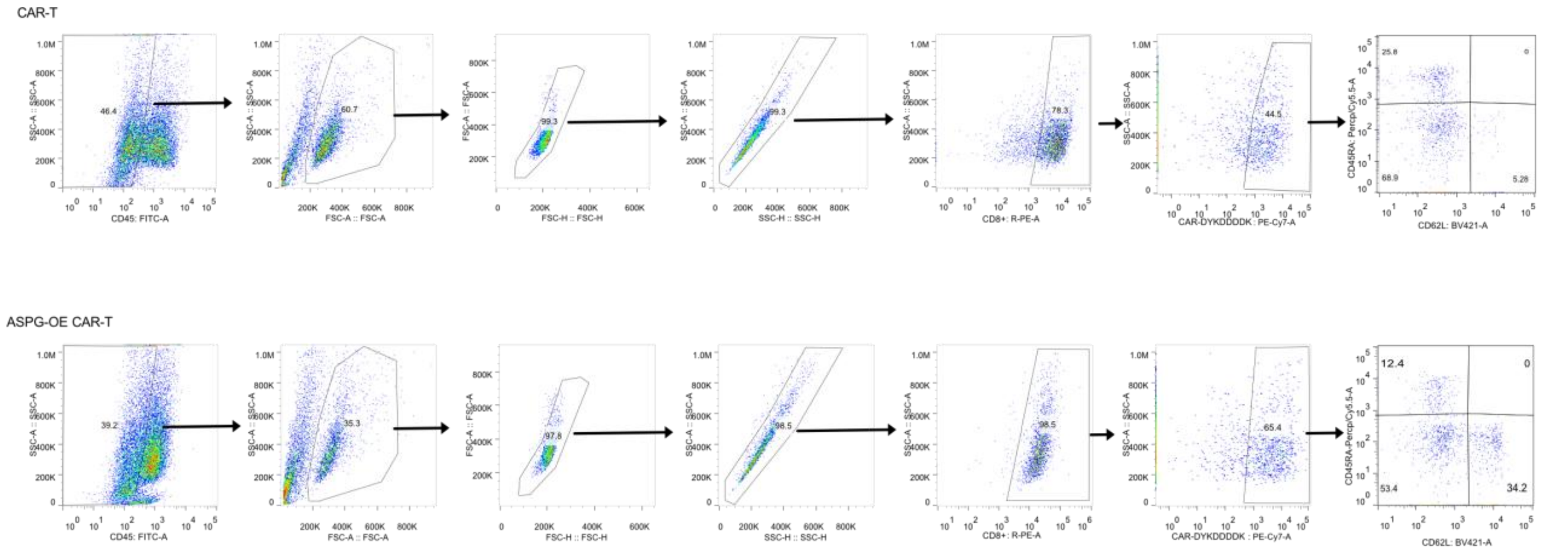

Figure S4: The gating strategy of Tcm (CD45RA^{-} CD62L^{+} CD8^{+} CAR^{+}), Tem (CD45RA^{-} CD62L^{-} CD8^{+} CAR^{+}), and Temra (CD45RA^{+} CD62L^{-}CD8^{+} CAR^{+}) of the resident CAR-T cells in the blood of mice at day 28 after receiving CAR-T and ASPG-OE CAR-T therapy.
